# Disentangling the Roles of Plant Water Status and Stem Carbohydrate Remobilization on Rice Harvest Index Under Drought

**DOI:** 10.1186/s12284-023-00631-6

**Published:** 2023-03-17

**Authors:** Sharad K. Dwivedi, Santosh Kumar, Mignon A. Natividad, Marinell R. Quintana, Viswanathan Chinnusamy, Amelia Henry

**Affiliations:** 1 ICAR - Research Complex for Eastern Region, Patna, Bihar 800014 India; 2 ICAR - Central Institute for Subtropical Horticulture, Lucknow, 226101 India; 3 International Rice Research Institute, Los Baños, Laguna, 4031 Philippines; 4 Division of Plant Physiology, ICAR - Indian Agricultural Research Institute, New Delhi, 110012 India

**Keywords:** Rice, Harvest index, Carbohydrate remobilization, Plant water status

## Abstract

**Background:**

Harvest index is an important component of grain yield and is typically reduced by reproductive stage drought stress in rice. Multiple drought response mechanisms can affect harvest index including plant water status and the degree of stem carbohydrate mobilization during grain filling. In this study, we aimed to dissect the contributions of plant water status and stem carbohydrate mobilization to harvest index. Pairs of genotypes selected for contrasting harvest index but similar biomass and days to flowering were characterized at ICAR-RCER, Patna, India and at IRRI, Philippines.

**Results:**

Multiple traits were related with harvest index across experiments, including mobilization efficiency at both sites as indicated by groupings in principal component analysis, and plant water status as indicated by direct correlations. Biomass-related traits were positively correlated with harvest index at IRRI but biomass was negatively correlated with harvest index at ICER-RCER, Patna. We observed that some pairs of genotypes showed differences in harvest index across environments, whereas other showed differences in harvest index only under drought. Of all time points measured when all genotypes were considered together, the stem carbohydrate levels at maturity were most consistently (negatively) correlated with harvest index under drought, but not under well-watered conditions. However, in the pairs of genotypes grouped as those whose differences in harvest index were stable across environments, improved plant water status resulted in a greater ability to both accumulate and remobilize stored carbohydrate, i.e. starch.

**Conclusion:**

By distinguishing between genotypes whose harvest index was improved across conditions as opposed to specifically under drought, we can attribute the mechanisms behind the stable high-harvest index genotypes to be more related to stem carbohydrate remobilization than to plant water status. The stable high-harvest index lines in this study (Aus 257 and Wanni Dahanala) may confer mechanisms to improve harvest index that are independent of drought response and therefore may be useful for breeding improved rice varieties.

**Supplementary Information:**

The online version contains supplementary material available at 10.1186/s12284-023-00631-6.

## Background

Harvest index, or the proportion of grain yield to the sum of grain yield, leaf, and stem biomass, is one of the main factors determining yield under drought (Passioura 1977). Harvest index has long been known to be affected by water availability (Araus et al. 2002), and has been advocated as a promising selection criterion for breeding since it tends to show simpler relationships with environmental conditions than does grain yield (Donald and Hamblin 1976). This study focuses on rice plant water status and stem carbohydrate remobilization, both of which have been summarized as important components of maximizing harvest index (Passioura and Angus 2010).

Although rice is considered to be one of the most drought susceptible crop species, genetic variation in plant water status has been established as evidenced by measures such as leaf water potential, stomatal conductance, and canopy temperature under drought (e.g. Mambani and Lal 1983; Sibounheuang et al. 2006; Cal et al. 2019; Melandri et al. 2020). Improved plant water status is generally concluded to increase harvest index under drought, although not all rice varieties with improved yield under drought exhibit high harvest index values (Anantha et al. 2016; Henry et al. 2015).

The level of stem carbohydrates remobilized to the grain can increase under water deficits especially at later growth stages, and rice exhibits a high degree of stem carbohydrate remobilization induced by drought compared with other grass species (Slewinski 2012). Management strategies based on mild water deficits at late reproductive stage or alternate wetting and drying (AWD) during vegetative stage have been outlined that improve stem carbohydrate remobilization and thereby the grain yield and water productivity (yield per amount of water consumed) of irrigated rice (Yang and Zhang 2010). However, the contribution of stem carbohydrate remobilization to drought tolerance of rice in terms of grain yield and harvest index under moderate to severe drought occurring at a range of growth stages has been less explored, although a number of studies relating stem carbohydrate remobilization to grain yield under drought have been reported in wheat (for example, Blum 1998; Ehdaie et al. 2006; El Habti et al. 2020).

Some preliminary evidence suggests a promising role for stem carbohydrate remobilization in the continued improvement of rice yield under drought. Sahbhagi dhan (IR74371-70-1-1), currently the most widely disseminated drought tolerant rice variety in South Asia (Dar et al. 2020), showed consistently higher harvest index values than other advanced drought breeding lines under drought, but not under well-watered conditions (Anantha et al. 2016). Another example is DRR dhan 42 (IR87707-445-B-B-B), a recently released drought tolerant variety in the background of IR64 that shows consistently lower canopy temperature than IR64 but not higher harvest index (Henry et al. 2015), and exhibits high levels of stem carbohydrates under drought (Torres et al. 2020). The trends for both Sahbhagi dhan and DRR dhan 42 suggest that increasing the level of stem carbohydrate remobilization across a range of soil moisture conditions could improve their yield even further. Similarly, YTH 183 is a rice breeding line that shows high harvest index across a range of soil moisture conditions which has been attributed to its remobilization of non-structural carbohydrates during grain filling, but the effects of YTH 183-derived QTLs on harvest index were not observed in all environments (Kato et al. 2011; Saito et al. 2021).

These examples illustrate the complex interaction between the roles of plant water status and stem carbohydrate remobilization in determining harvest index under drought that are not easily distinguished. Even in simulation models, drought stress effects on carbohydrate remobilization have not been frequently incorporated since drought can affect both sink activity (i.e. grain formation) and source activity (such as photosynthesis and plant water status; Stella et al. 2016). Some genotypes may exhibit higher harvest index under drought due to drought-response mechanisms related to plant water status that improve grain yield, and other genotypes may exhibit higher harvest index under drought due to their general ability to remobilize stem carbohydrates regardless of the soil moisture status. In this study, we aimed to dissect the different potential mechanisms behind higher harvest index under drought. We investigated genetic variation for rice harvest index under drought and the related stem traits on a biochemical (in terms of non-structural carbohydrates) and gravimetric basis (based on changes in stem dry weight), in comparison with plant water status measured indirectly by stomatal conductance and canopy temperature (Jones 2007). We hypothesized that plant water status and stem carbohydrate remobilization would show complementary effects on harvest index (i.e. that either one or the other trait would dominate for a given genotype), and that genotypes with better plant water status under drought would exhibit less stem carbohydrate remobilization during grain filling.

## Methods

### Genotypes and Field Experiments

To investigate traits related to high harvest index under drought among traditional rice varieties, six pairs of genotypes (Table 1) were identified from previous drought studies on indica and aus Genebank material (Torres et al. 2013; Liao et al. 2022). In order to pinpoint effects of stem carbohydrate mobilization and minimize the effects of differences in biomass and flowering time on harvest index (HI) and drought response, the genotypes within each pair were selected based on their similar above-ground biomass and days to flowering (DTF) but contrasting harvest index across at least four separate experiments including drought and well-watered treatments. A seventh pair of improved varieties that are well established as drought susceptible and drought tolerant—IR64 and Sahbhagi dhan (IR74371-70-1-1), respectively (Table 1)—were included as checks. The genotypes were classified as “High HI” or “Low HI” based on the previously observed relative trends within each pair.

**Table 1:** Description of the genotype pairs selected for this study with similar shoot biomass and time to flowering but contrasting harvest index

Pair	Genotype	IRGC #	HI class	Description/reference
1	Jabor Sail	66,831	High	Aus; identified for high grain yield under drought (Torres et al. 2013)
1	Tchampa	32,362	Low	Aus
2	Dular	117,266	High	Aus; previously noted for deep root growth, low canopy temperature, and high drought response index (Henry et al. 2011)
2	Santhi Sufaid 207	28,212	Low	Aus
3	Camponi Sml	50,640	High	Indica
3	Gul Murali	66,792	Low	Indica
4	ARC 10,955	12,683	High	Aus; high yield under drought and high deep root dry weight (Liao et al. 2022)
4	Soloi	37,598	Low	Aus
5	E Zi 124	70,215	Low	Indica
5	Wanni Dahanala	15,721	High	Indica
6	Aus 257	29,049	High	Aus; high yield under drought and well-watered conditions (Liao et al. 2022)
6	DZ78	117,610	Low	Aus
7	IR64		Low	Indica; previously a mega-variety in Asia, drought susceptible (Mackill and Khush 2018)
7	IR74371-70-1-1		High	Indica; released as Sahbhagi dhan in India, BRRI dhan 56 in Bangladesh, Sukha dhan 3 in Nepal; exhibited consistently high harvest index under drought (Anantha et al. 2016)

A total of 14 experiments (seven drought and seven well-watered) were conducted to evaluate traits related to harvest index in these genotype pairs across three kharif (wet) seasons of 2017–2019 at the ICAR Research Complex for Eastern Region, Patna, Bihar, India (“Patna”; 25°30′ N, 85°15′ E, 52 masl), and the 2017–2018 wet seasons and 2018–2019 dry seasons at the International Rice Research Institute, Los Baños, Laguna, Philippines (“IRRI”; 14°10′ N, 121°15′ E, 21 masl). The inclusion of these two study sites provided a more controlled drought environment (as provided by a rainout shelter) and capacity for more physiological measurements (IRRI), as well as an environment representative of those frequently faced by rice farmers in drought-prone regions (Patna). All 14 genotypes were included in each experiment, except in the 2017WS and 2018DS experiments at IRRI in which three to four genotypes were missing due to limitations in seed availability. All experiments were laid out in a randomized complete block design (RCBD) with four replicates per genotype.

Twenty one to 27 (Patna) and 17 (IRRI) days old seedlings were transplanted in the main experimental field with row to row spacing of 20/25 cm and plant to plant spacing of 15/20 cm (Patna/IRRI). The plot size was 4 m^2^ in Patna experiments and 3–3.75 m^2^ in IRRI experiments. In Patna, fertilizer was applied at 120, 60 and 40 kg ha^−1^ N, P_2_O_5_ and K_2_O, respectively, with P_2_O_5_ and K_2_O applied as basal fertilizer and nitrogen applied in three equal measures (as basal, at maximum tillering, and at panicle initiation) in the well-watered treatment. In the drought stress treatment, nitrogen was applied in two equal measures (as basal and at maximum tillering). At IRRI, complete fertilizer (14N-14P-14 K) was applied at 50 kg N ha^−1^ as basal application, and a topdressing of 50 kg N ha^−1^ ammonium sulfate was applied at maximum tillering. All experiments were carried out in an open field, except for the IRRI drought experiments which were planted in a 7 m x 20 m automated rainout shelter. The soil type at Patna was a Eutric Fluvisol with a pH of 7.4 and bulk density at 25–30 cm depth of 1.48 g cm^−3^, and at IRRI was an Isohyperthermic Typic Hapludalf with a pH of 7.5 and bulk density at 25–30 cm depth of 1.09 g cm^−3^.

The well-watered control experiments were kept continuously flooded after transplanting until 25 days before harvest. The drought-stress experiments were kept continuously flooded for 57–62 days after sowing at Patna and 43–49 days after sowing at IRRI, and then irrigation was withdrawn to initiate the drought stress treatment during reproductive stage. Once the drought stress treatments were initiated, the Patna drought experiments were carried out as rainfed experiments, whereas the IRRI drought experiments were rewatered once or twice per season by flash flooding to alleviate the severe drought stress symptoms exhibited. Soil moisture levels were monitored by tensiometers installed at a depth of 30 cm in each experiment as well as water table tubes installed to a depth of 1 m.

### Agronomic and Physiological Traits

Days to 50% flowering, grain yield (g m^−2^, normalized for a 14% grain moisture content), straw biomass (g m^−2^), and harvest index [grain yield/(straw biomass + grain yield)] were recorded in all experiments. The grain yield and straw biomass were harvested from an area of 4 m^2^ in Patna experiments and 1–1.5 m^2^ in IRRI experiments. Most genotypes (except IR64 and IR74371-70-1-1) exhibited lodging towards maturity—especially in the well-watered experiments. Lodged plants were tied to bamboo stakes until maturity in the IRRI experiments but not in the Patna experiments.

Stem samples (10 tillers from 1 hill per plot) were taken in all experiments to determine stem dry weight (all experiments) and stem length (all Patna experiments; 2018WS and 2019DS IRRI experiments). Stem samples were taken weekly in the IRRI 2017WS and 2018DS experiments and at anthesis and maturity in all Patna experiments and the 2018WS and 2019DS IRRI experiments. In Patna, the tillers were separated into stems and leaves, dried in an oven at 80 °C for four hours, and then dried at 60 °C until a constant dry weight was recorded. At IRRI, leaves were separated from the stem with the leaf sheath intact, and then the tillers were oven-dried at 70 °C for 3 days.

Specific stem weight (SSW), stem reserve mobilization (SRM), and mobilization efficiency (ME) were calculated as follows:

$${\text{SSW}}: \frac{ave\, stem\; mass}{{ave \;stem\; length}}$$

$${\text{SRM}}:\frac{SSW\;anthesis - SSW\;maturity}{{SSW\;anthesis}} \times 100$$

$${\text{ME}}:\frac{Stem\; mass \;anthesis - Stem\; mass\; maturity}{{Stem\, mass\; anthesis}} \times 100$$

Carbohydrates were measured by colorimetry using the anthrone reagent for the stem samples from all plots in the 2017WS IRRI experiment and 10% of the stem samples in the remaining IRRI experiments. Values obtained for the IRRI 2017WS stem samples were used to create a model (Hone Pty Ltd; Newcastle, Australia) to predict the non-structural carbohydrate content by Fourier transform infrared spectroscopy (FTIR; PerkinElmer Spectrum Two). Before creating the model, 25% of the data set was automatically selected in the Hone platform and used as a validation test for a more robust test on the model created. Pre-processing of the data set was also automatically done before building a model to check which different models will be best suited to the data set. From the succeeding seasons, values obtained from the colorimetric assay were added into the model to develop a more efficient and accurate model. FTIR measurements were used in the 2018DS, 2018WS, and 2019WS experiments to maximize the number of samples measured in a lesser amount of time.

For the IRRI colorimetric analysis on stem tissue, oven dried stems were placed directly into a mill (Marathon Electric, Mexico) and ground (40 mesh). Sugar soluble in ethanol was extracted from a subsample of 200 mg ground tissue according to Conocono et al. (1998). Absorbance was determined at 620 nm (UV-1800 Spectrophotometer, Shimadzu Corp.) and the concentration of each sample was calculated in reference to a glucose standard curve (Dextrose, Anhydrous, Fischer Scientific Co.). For the FTIR spectroscopy, ground samples kept in a 50 °C oven were allowed to reach room temperature before taking FTIR measurements. A small amount of ground sample—just enough to cover the FTIR crystal—was scanned in 4 accumulations ranging from wave numbers of 4000–400 cm^−1^ and with a resolution of 4 cm^−1^. The FTIR spectrum was recorded as absorbance against wave number (cm^−1^). Values obtained in the FTIR measurements were run in the Hone Ag platform to predict the soluble sugar content.

Canopy temperature (MI-210; Apogee Instruments, UT, USA; 3 sensors per plot at a 1-m height and 45° angle) and normalized difference vegetation index (NDVI; Crop Circle ACS 470 sensor, Holland Scientific, NE, USA) were measured on sunny days in the IRRI drought experiments from a semi-automated sensor rack that rolled along the rails of the rainout shelter. Readings were taken along the length of each plot on multiple dates per experiment.

Gas exchange measurements were conducted using a portable infrared gas analyzer (LI-6400 Model, LICOR*,* USA) on the individual anthesis dates in each plot (Patna and IRRI) and also together on the same date (70–91 DAS in 2018WS-2019DS) at IRRI. The gas exchange measurements were done on fully expanded flag leaves at Patna and on the second or third leaves at IRRI, between 9:00 h and 11:00 h. The chamber was set to maintain light levels at 1000 μmol m^−2^ s^−1^ in the Patna experiments and to maintain light levels at 1500 μmol m^−2^ s^−1^, CO_2_ levels at 400 ppm, leaf temperature set at the ambient temperature at the time of measurement in Patna and 28 °C at IRRI, and relative humidity at 65% at IRRI.

### Statistical Analysis

To confirm that the genotypes within each HI class pair exhibited different HI values in this study but similar DTF and biomass values for which they were selected, Analysis of Variance (ANOVA) was used in comparisons of genotypic means for HI, straw biomass, and ME in each experiment (season/treatment), following those assumptions of ANOVA. Tukey’s HSD was the post hoc analysis used to determine the differences between genotypic means for each trait. Letter groups were assigned to each genotypic mean, and means with the same letter were considered as not statistically different. The analysis was performed using a Statistical Tool for Agricultural Research (http://bbi.irri.org) in combination with the R program *agricolae* package. To determine which pairs showed the most stable differences in HI across experiments, we counted the total number of experiments in which the high-HI genotype showed significantly higher HI than the low-HI genotype, and we determined the proportion of those experiments that were from well-watered experiments (i.e. the number significant well-watered experiments divided by the number of significant drought stress experiments).

For the other agronomic traits measured (DTF, plant height, tiller number, and grain yield), we conducted a multi-environment analysis with the two sites and treatments analyzed separately using R v. 4.0.3 (https://www.R-project.org/). A one-stage analysis was conducted using Linear Mixed Effects Models (*lme4*) with Randomized complete block design for each experiment. Genotype means (BLUEs) across experiments was obtained, followed by a pairwise analysis using emmeans and Tukey’s test to identify differences among genotypes.

To identify which physiological traits were consistently correlated across multiple experiments with HI in IRRI and Patna experiments, we used both principal component analysis (PCA) and correlations. One PCA was conducted for each site with both drought and well-watered experiments included, using only traits that were conducted in all experiments per site. The PCA was run using *prcomp* in R v.4.2.1 with missing data imputed using *missMDA* and clustering of data done with *hclust*.

Data from each individual experiment (genotype means for Patna experiments and replicated data for IRRI experiments) were subjected to Pearson’s correlation analysis (*correlation*). For the IRRI experiments, the maximum canopy temperature and the corresponding NDVI values on that date were used in the correlation analysis. Mean seasonal canopy temperature and NDVI values were calculated (*doBY*) to compare genotypes within each pair by ANOVA and LSD (*agricolae*). All statistical analysis included all genotypes grown in each season to be consistent with the experimental design, with replication effect taken into account; none were excluded from the statistical analysis but Pair 4 was excluded from the figures shown since it did not exhibit the HI class differences for which it was selected to be included in this study.

## Results

### Response of HI Class Pairs to the Study Environments

**Fig. 1:**
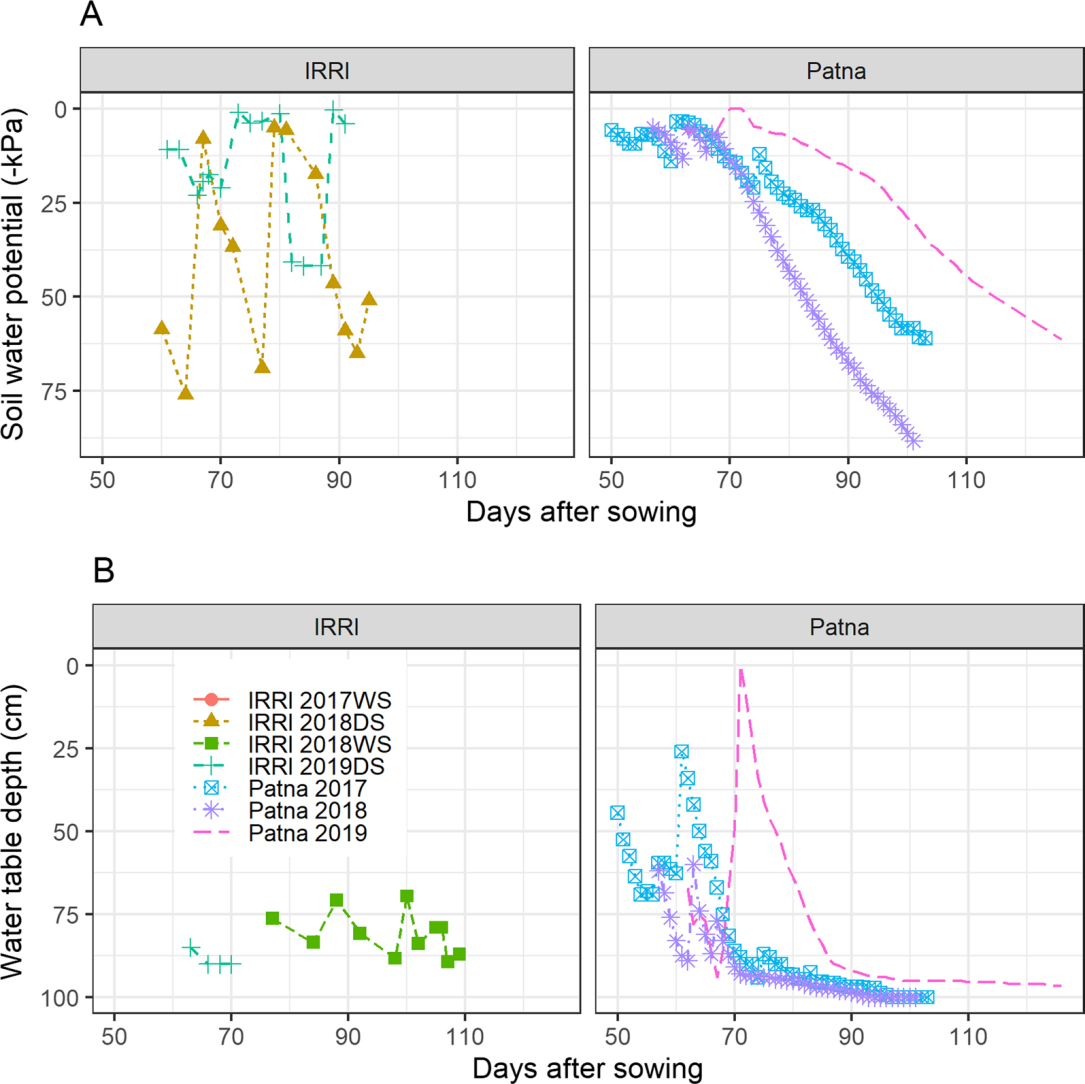
Drought stress characterization across experiments. **A** Soil water potential at a depth of 30 cm as measured by tensiometer, **B** water table depth. Drought stress treatments were initiated at 57–62 days after sowing at Patna and 43–49 days after sowing at IRRI

**Fig. 2:**
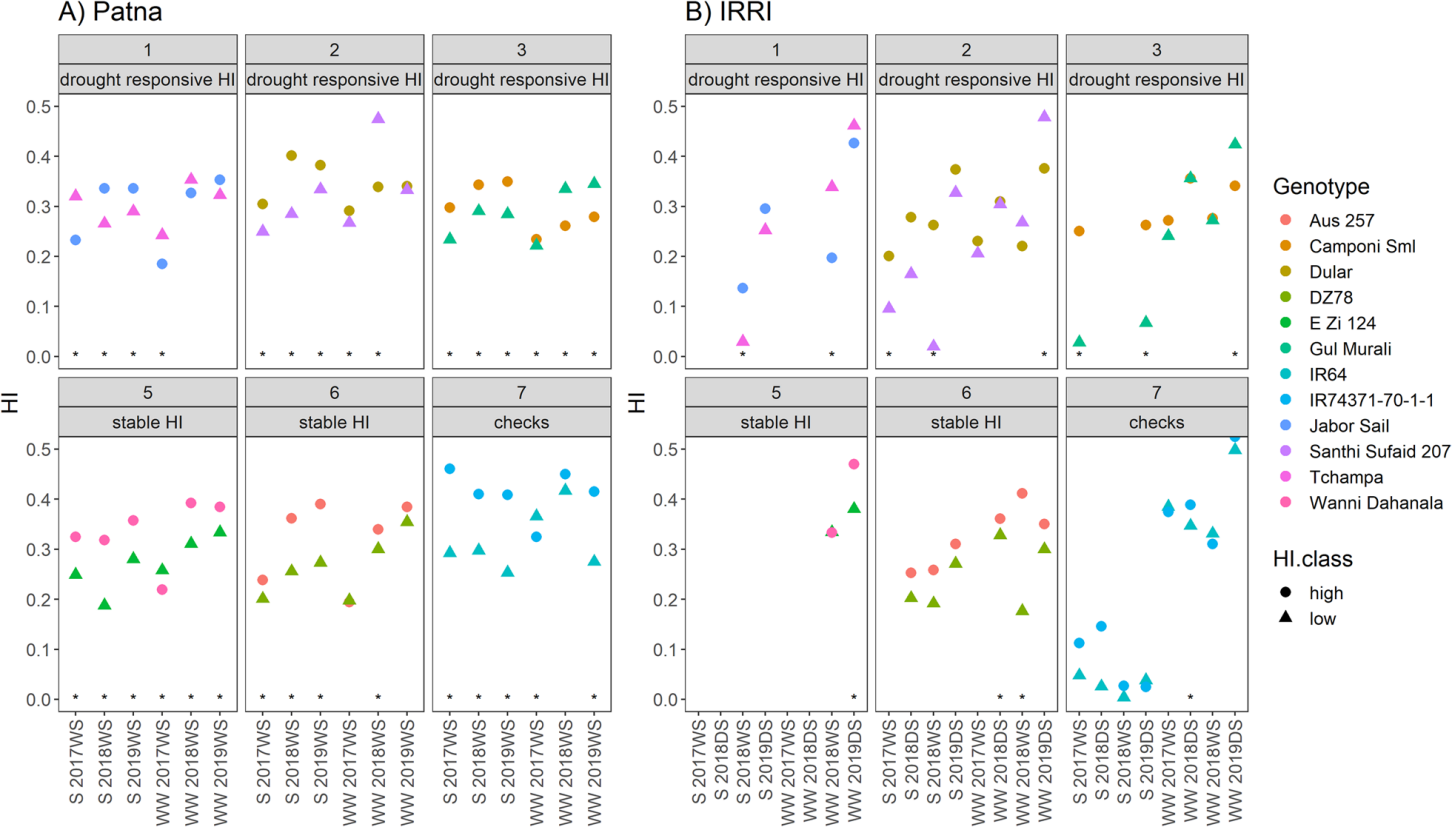
Harvest index across experiments under drought stress (S) and well-watered (WW) conditions at **A** Patna and **B** IRRI. The boxes at the top of each panel indicate the pair ID as well as the classification of the HI response as stable across treatments (stable HI) or more prevalent under drought stress (drought-responsive HI). Significant differences between genotypes within a pair (based on different letter groups in according to Tukey’s test) are indicated by *. WS: wet season, DS: dry season. The treatment, year, and season of each experiment are shown on the x-axis

The soil in the drought stress experiments at IRRI generally tended to dry more quickly and the soil moisture fluctuated more as a result of re-watering, compared to the Patna experiments which showed a gradual soil drydown throughout the drought stress treatments (Fig. 1). Climatic conditions between the two sites were similar, although the mean seasonal temperatures at IRRI were slightly higher than at Patna (Additional file 1: Table S1). All genotype pairs generally exhibited the expected differences in harvest index for which they were selected (Fig. 2), but these differences were not observed in the case of Pair 4 (ARC 10,955 and Soloi; Additional file 1: Fig S1). Out of all experiments in Patna and at IRRI in which the high-HI genotype showed significantly greater HI than the low-HI genotype, the proportion that were well-watered ranged from 0 to 0.2 for Pairs 1, 2, and 3 which were thus classified as “drought-responsive HI pairs”. The proportion was 1.0 (indicating equal numbers of well-watered and drought experiments in which the high-HI genotype showed significantly greater HI) for Pairs 4 and 5, which were thus classified as the “stable HI” pairs. The classifications “drought-responsive HI” and “stable HI” (Fig. 2) were thus used for subsequent analysis. We did not assign a classification to Pair 7 since it was selected as drought tolerant and drought susceptible check varieties and not for previously showing similar biomass and DTF.

Among other agronomic traits measured, the genotypes within a pair showed similar days to flowering, tiller number at maturity, plant height, and grain yield (Additional file 1: Table S2), with some exceptions especially for tiller number in the Patna drought stress experiments and for DTF in the Patna well-watered experiments. Straw biomass values at Patna were consistently higher than those at IRRI in both treatments (Additional file 1: Fig S2), and no consistent trend between the high and low HI genotypes of each pair was observed. ME values were mostly positive but ranged to negative values in some of the drought experiments, and no consistent trend between the high and low HI genotypes of each pair was observed (Additional file 1: Fig. S3).

Both stem soluble sugar (Additional file 1: Fig. S4) and starch (Additional file 1: Fig. S5) values increased until around anthesis and then decreased by the time of maturity. Few consistent differences between high and low HI genotypes of each pair were observed across experiments for stem soluble sugar and starch at anthesis and maturity (Additional file 1: Figs. S6 and S7), except for Pair 2 in which the high HI genotype showed significantly lower stem starch content at maturity than the low HI genotype in two drought experiments, and Pair 7 in which the high HI genotype showed significantly lower stem starch content at maturity than the low HI genotype in three well-watered experiments.

The canopy temperature of the high HI genotype was generally lower (Additional file 1: Fig S8). The high and low HI genotypes of Pair 2 (Dular and Santhi Sufaid 207), Pair 5 (E Zi 124 and Wanni Dahanala) and Pair 3 (Camponi Sml and Gul Murali) showed significant differences in mean canopy temperature in multiple experiments (Additional file 1: Table S4). The NDVI of the high HI genotype was higher across experiments in some cases (Additional file 1: Fig S9), with the high HI genotype of Pair 3 (Camponi Sml and Gul Murali) and Pair 5 (E Zi 124 and Wanni Dahanala) significantly higher in three of the IRRI drought experiments (Additional file 1: Table S5). Few consistent differences between high and low HI genotypes of each pair were observed across experiments for photosynthesis rates and stomatal conductance at anthesis at Patna and IRRI (Additional file 1: Figs. S10 and S11), except for Pair 1 (Jabor Sail and Tchampa) in which the high HI genotype showed higher photosynthesis rates across all Patna experiments, and Pair 2 (Dular and Santhi Sufaid 207) in which the high HI genotype showed higher stomatal conductance across both IRRI drought experiments.

### Traits Correlated with Harvest Index

**Fig. 3:**
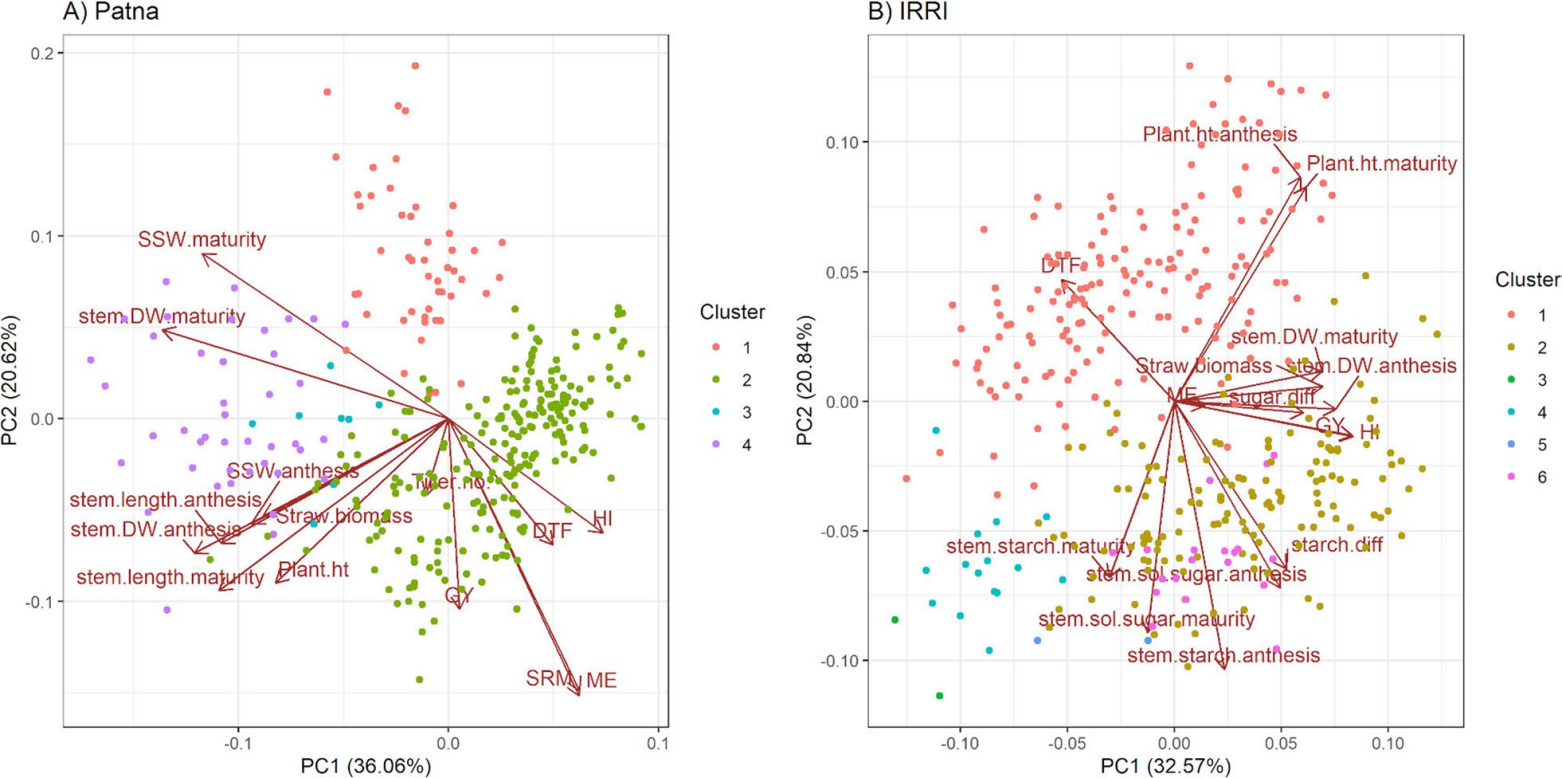
Principal component analysis of trait measured across experiments at **A** Patna and **B** IRRI. Principal component 1 is shown on the x-axis and principal component 2 is shown on the y-axis. The percentage values indicate the variation explained by each principal component. DTF: days to flowering, DW: dry weight, GY: grain yield, HI: harvest index, ME: mobilization efficiency, Plant.ht: plant height, SRM: stem reserve mobilization, SSW: specific stem weight

In the PCA on all traits measured across experiments, the first two PCs explained 55% of the variation in the Patna experiments and 71% of the variation in the IRRI experiments (Additional file 1: Table S3). Clustering of the data from the Patna experiments was largely based on growth season; cluster 1: 2017WS drought experiment, cluster 2: 2018WS and 2019WS experiments (both treatments), cluster 3: 2017WS well-watered experiment Pair 7 (checks), and cluster 4: 2017WS well-watered experiment (Fig. 3A). Clustering of the data from the IRRI experiments generally separated the wet season (cluster 1) from the dry season experiments (cluster 2; Fig. 3B). Traits that grouped with harvest index in the PCA biplot for Patna were DTF, SRM, ME, and grain yield (Fig. 3A). Traits that grouped with harvest index in the PCA biplot for IRRI were stem dry weight at anthesis, stem dry weight at maturity, the difference in soluble sugar content between anthesis and maturity, ME, straw biomass, and grain yield (Fig. 3B).

**Fig. 4:**
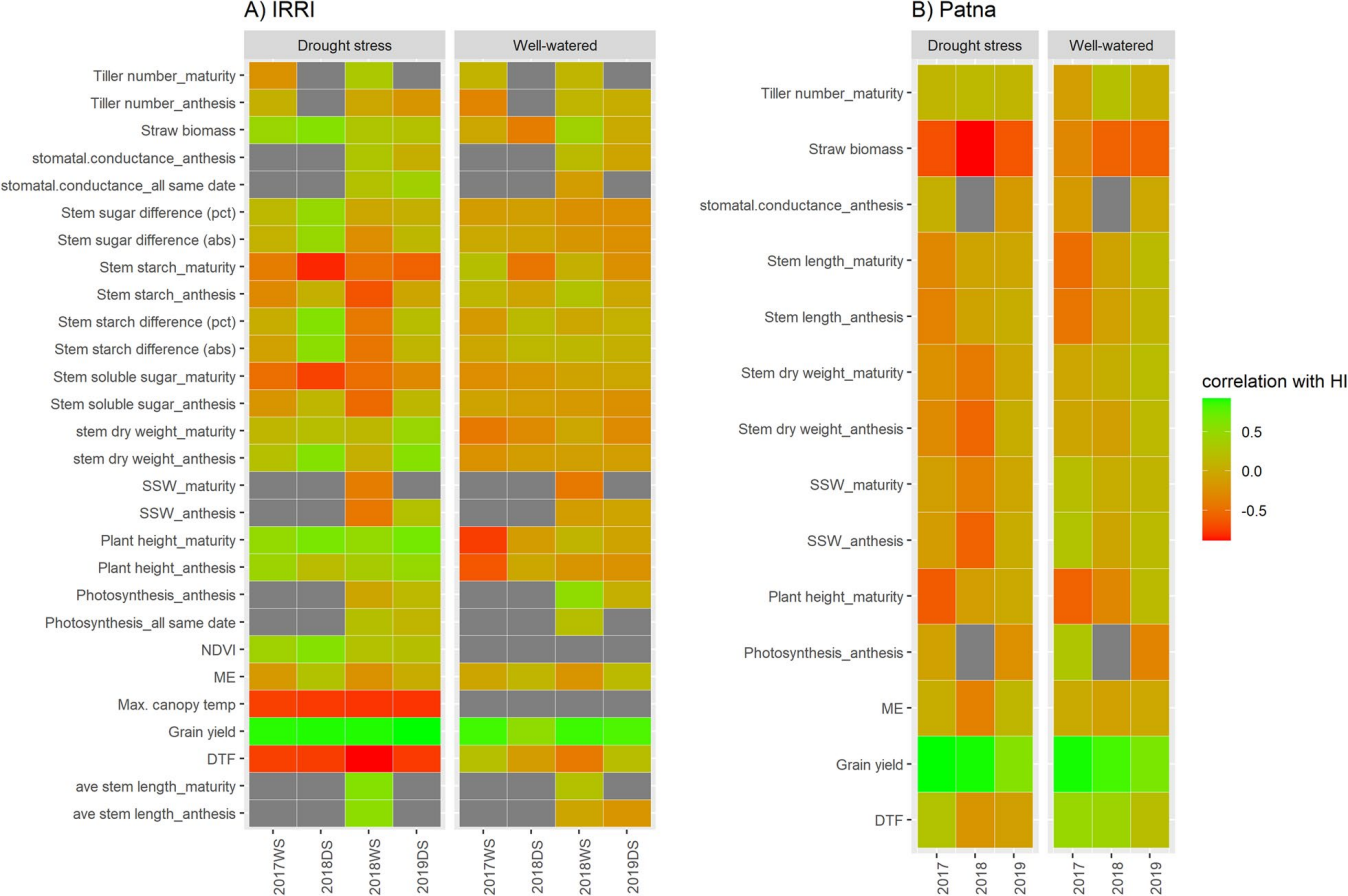
Correlations between harvest index and the physiological and agronomic traits measured at **A** IRRI and **B** Patna. Abs: absolute change, DTF: days to flowering, HI: harvest index, ME: mobilization efficiency, NDVI: normalized difference vegetation index, pct: percent change, SSW: specific stem weight. The year and season of each experiment are shown on the x-axis

Within individual experiments, significant correlations with harvest index were consistently positive for grain yield across all experiments (Fig. 4, Additional file 2: Table S6 and Additional file 3: Table S7), but the direction of correlations between harvest index and other agronomic traits differed between Patna and IRRI. Whereas biomass, plant height, stem dry weight, and NDVI were positively correlated with harvest index in IRRI drought experiments (Fig. 4A, Additional file 2: Table S6), straw biomass was negatively correlated with harvest index across all Patna experiments and stem dry weight was negatively correlated with harvest index in multiple Patna drought experiments (Fig. 4B, Additional file 3: Table S7). Days to flowering was negatively correlated with harvest index across IRRI drought experiments (Fig. 4A, Additional file 2: Table S6) and was positively correlated with harvest index across Patna well-watered experiments.

In terms of physiological traits, the maximum canopy temperature measured in each drought experiment was negatively correlated with harvest index, while stomatal conductance showed positive correlations with harvest index across multiple drought stress experiments (Fig. 4A, Additional file 1: Table S6). The stem carbohydrate content (soluble sugar and starch) at maturity stood out as the only resource remobilization-related trait to show significant direct (negative) correlations with harvest index; ME, leaf carbohydrate levels, stem carbohydrate levels at anthesis, and the difference in stem carbohydrate levels from anthesis to maturity (relative or absolute) were not consistently correlated with harvest index (Fig. 4A, Additional file 2: Table S6). Stem resource remobilization traits based on biomass (SSW, ME) were not correlated with HI within the individual experiments (Fig. 4, Additional file 2: Table S6 and Additional file 3: Table S7).

### The Contributions of Plant Water Status and Stem Carbohydrate Remobilization to Harvest Index

**Fig. 5:**
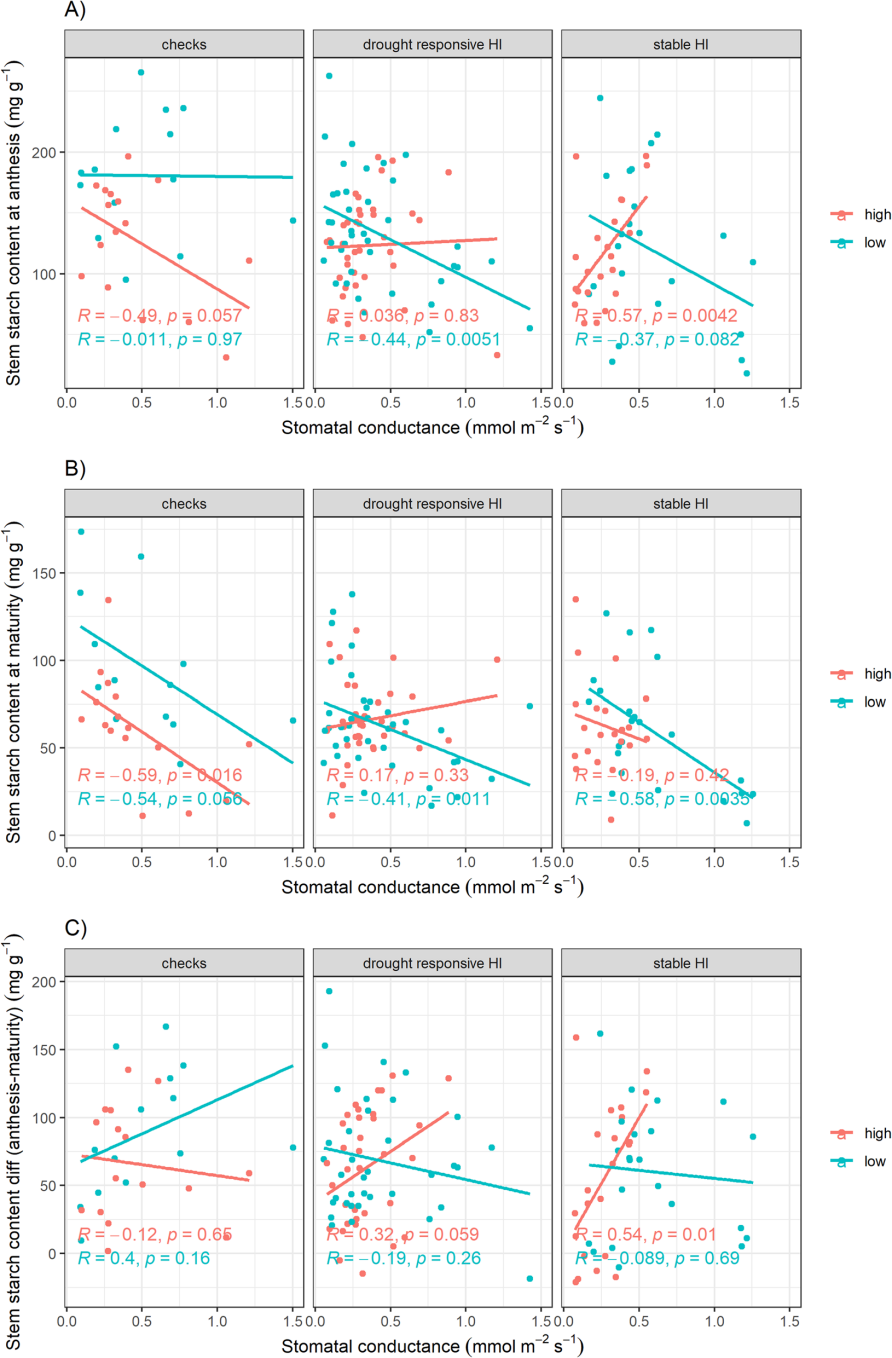
Relationships between plant water status (as indicated by stomatal conductance at anthesis) and stem carbohydrate remobilization (as indicated by stem starch content) across IRRI drought and well-watered experiments. Correlations are shown between stomatal conductance and stem starch levels at anthesis (**A**), maturity (**B**), and the difference between anthesis and maturity (**C**) among groups whose difference in harvest index between high-HI and low-HI genotype pairs appeared to be stable (Pairs 1, 2, and 3) or drought-responsive (Pairs 5 and 6). The checks were IR74371-70-1-1 (high-HI) and IR64 (low HI)

To better understand the individual contributions of plant water status (as reflected by canopy temperature and stomatal conductance) and stem carbohydrate remobilization (in terms of stem soluble sugar and starch content and ME) under drought stress, we considered correlations within the groups showing HI differences that were stable or drought responsive (as indicated in Fig. 2). The greatest distinction between the stable HI group and the drought responsive HI group was in terms of stem starch content: when stomatal conductance was higher at anthesis, the stable high-HI genotypes showed higher stem starch levels at anthesis (Fig. 5A) and a greater difference in stem starch content between anthesis and maturity (Fig. 5C). In contrast, the drought-responsive high-HI lines showed lower levels of stem starch (Fig. 5B) and soluble sugar (Additional file 1: Fig. S12B) at maturity with increased stomatal conductance at anthesis. A trend of lower levels of stem soluble sugar at anthesis as stomatal conductance decreased (when all genotypes were measured on the same date) was observed in all groups of genotypes (Additional file 1: Fig. S12A). A negative relationship between stem starch levels at maturity and canopy temperature in the drought experiments was most evident for the drought susceptible check, IR64 (Additional file 1: Fig. S12C). Between stomatal conductance at anthesis and ME, a negative trend was observed only for the drought tolerant check, IR74371-70–1-1 at Patna (p = 0.063; Additional file 1: Fig. S13).

## Discussion

Given the inconsistent harvest index values of relatively recently-developed rice cultivars for drought-prone environments (Henry et al. 2016; Anantha et al. 2016; Saito et al. 2021), we aimed to identify rice genotypes that showed consistently high harvest index values across environments with a range of soil moisture levels. We therefore designed this study to compare the harvest index within pairs of genotypes that previously showed similar time to flowering and biomass but contrasting (high or low) harvest index, to focus on the mechanisms behind harvest index rather than on effects of phenology or allometry. Through this study, it became apparent that some of pairs showed differences in harvest index only under drought stress (drought-responsive HI), whereas other pairs showed more stable differences in harvest index across both drought and well-watered conditions (stable HI; Fig. 2). The importance of high harvest index under well-watered conditions has previously been advocated as an important component of maintaining stable yields in rainfed systems, together with early flowering for drought escape and maintained growth and leaf water status under drought (Jearakongma et al. 1995; Fukai et al. 1999).

Although we hypothesized that either plant water status or stem carbohydrate remobilization would dominate the effects on harvest index within a given genotype pair, our results indicate a more complex interaction between the two mechanisms as well as the importance of short-term and longer-term stem carbohydrate sources (i.e. soluble sugars *vs* starch). When all genotypes were considered together, both stem starch and soluble sugar levels at maturity (Fig. 4A, Additional file 2: Table S6) as well as ME (Fig. 3) appeared to explain the variation in harvest index under drought to a large degree. However, when the drought-responsive HI and stable HI pairs were considered separately, the relationships among these traits were not consistent. We consider the stomatal conductance and canopy temperature measurements measured at anthesis as a general indicator of plant water status that could subsequently affect stem carbohydrate remobilization by maturity. In the stable HI-genotypes, better plant water status appeared to facilitate greater stem carbohydrate accumulation as well as a greater degree of remobilization, as evidenced by the higher stem starch levels at anthesis with increasing stomatal conductance of the stable high-HI genotypes (Fig. 5A) and the greater difference in stem starch content between anthesis and maturity (Fig. 5C). In contrast, the drought-responsive high-HI genotypes appeared to have impaired ability to remobilize stem carbohydrates based on their higher levels of stem starch (Fig. 5C) and soluble sugar (Additional file 1: Fig. S12B) at maturity as stomatal conductance decreased. Both genotype groups exhibited lower levels of stem soluble sugar at anthesis as stomatal conductance decreased (Additional file 1: Fig. S12A); this suggests that reduced plant water status may trigger more of a short-term remobilization that is common across genotypes, in contrast to the longer-term remobilization patters associated with starch levels and ME. The drought-responsive high-HI genotypes may have relied on drought avoidance mechanisms to maintain their high harvest index under drought. Therefore, the stable high-HI genotypes Aus 257 and Wanni Dahanala which showed high harvest index even in the well-watered experiments may be good sources of mechanisms for high harvest index that are independent of drought response mechanisms and might be useful in breeding for stable harvest index improvement across environments. Specifically, Aus 257 and Wanni Dahanala showed lower stem starch levels at maturity than the drought tolerant check IR74371-70–1-1 (Additional file 1: Figs. S5 and S7), suggesting that they could contribute beneficial mechanisms to the drought breeding pool.

The interaction between plant water status and stem carbohydrate remobilization has been addressed under mild terminal water deficit during grain-filling with high- and normal-N treatments by Yang et al. (2001). In that study, more negative mid-day leaf water potential in a high-N treatment slowed stem carbohydrate remobilization, which appears to be consistent with the trends observed in the stable high-HI genotypes in this study. However, Fu et al. (2011) cited high stem carbohydrate levels at anthesis as important in determining irrigated rice sink strength (including enzyme activity in inferior spikelets, endosperm cell number and size, and grain filling rate). The different environmental conditions (soil moisture levels, drydown rates, etc.) among studies likely explain some of the differences observed in the role of stem carbohydrate remobilization in determining harvest index. Okamura et al. (2018) concluded that translocation efficiency may have a stronger effect on grain yield than non-structural carbohydrate accumulation, which is in agreement with our study in which the stem carbohydrate levels at maturity, rather than the difference between stem carbohydrate levels at anthesis and maturity, were most correlated with harvest index. Rodrigues et al. (2019) presented evidence that source strength can be resilient to drought. Our study suggests that these trends may depend on the genotypes studied and the environmental conditions. Furthermore, the reproductive stage drought stress (initiated around panicle initiation) likely impaired fertility, thereby reducing sink strength especially in the low-HI genotypes of the drought-responsive HI pairs.

Another notable result from this study was the role of biomass, which Donald and Hamblin (1976) had reported as not generally correlated with harvest index but which was positively correlated with harvest index under drought and well-watered conditions at IRRI (Fig. 4A, Additional file 2: Table S6) and negatively correlated with harvest index under drought and well-watered conditions in Patna (Fig. 4B, Additional file 3: Table S7). Since these differences in trends were observed in both soil moisture treatments, some environmental characteristic (temperature, humidity, light levels, soil fertility, or soil physical properties) other than drought severity may have affected the relationship between harvest index and biomass, and this may be related to the higher biomass in Patna experiments (Additional file 1: Fig. S2).

The different correlations with harvest index between study sites indicates a possibility that different trends in stem carbohydrate remobilization observed at IRRI may be occurring in Patna, although stem carbohydrate levels were only measured at IRRI. More research, including characterization of stem carbohydrate remobilization in target drought-prone environments such as Patna as well as identification of potential donor genotypes with an effect on harvest index under well-watered conditions, is necessary.

## Conclusions

To improve rice harvest index under drought stress, it is likely that multiple physiological mechanisms should be targeted. For example, both better plant water status and better stem carbohydrate remobilization may provide a greater benefit to rice harvest index under drought compared with improvement of only one of those mechanisms. However, it is difficult to disentangle these two mechanisms because both can affect harvest index, and because drought stress tends to increase stem carbohydrate remobilization in rice. Therefore, in this study, we took the approach of including the well-watered treatment in our evaluation of genotypes for stem carbohydrate remobilization in order to pinpoint genotypes with improved stem carbohydrate remobilization that is independent of drought response mechanisms.

We hypothesized that there would be compensation between plant water status and stem carbohydrate remobilization mechanisms related to rice harvest index, and this appeared to be the case to some extent for the drought-responsive high HI genotypes in this study. However, the genotypes with the most stable and high harvest index appeared to exhibit more of a synergistic effect in which improved plant water status resulted in a greater ability to both accumulate and remobilize starch. These results suggest that improving rice harvest index under drought via both plant water status and stem carbohydrate mobilization would be possible if optimal donor genotypes are identified. Further studies dissecting more detailed mechanisms and the related genetics behind the stable high-HI lines in this study may be useful for breeding improved rice varieties.

## Supplementary Information

**Additional file 1.** **Table S1.** Climatic conditions across each season of study. **Table S2.** Differences in agronomic traits between genotypes in each pair selected for this study. **Table S3.** Variance explained by each principal component (PC) from the principal component analysis from Patna and IRRI. **Table S4.** Mean seasonal canopy temperature differences (IRRI drought stress experiments) grouped by pairs of genotypes with differing HI under drought but similar biomass and time to flowering. **Table S5.** Mean seasonal NDVI differences (IRRI drought stress experiments) grouped by pairs of genotypes with differing HI under drought but similar biomass and time to flowering. **Fig. S1.** Genotype Pair 4 did not exhibit the expected HI differences across trials for which the genotypes were selected. **Fig. S2.** Straw biomass across experiments under drought stress (S) and well-watered (WW) conditions at A) Patna and B) IRRI. **Fig. S3.** ME ((Stem mass anthesis -Stem mass maturity)/Stem mass anthesis ×100) across experiments at IRRI at ICAR-Patna under A) drought stress and B) well-watered conditions. **Fig. S4.** Stem soluble sugar content throughout the season in drought (A-B) and well-watered (C-D) experiments at IRRI. **Fig. S5.** Stem starch content throughout the season in drought (A-B) and well-watered (C-D) experiments at IRRI. **Fig. S6.** Stem soluble sugar content across IRRI experiments at A) anthesis and B) maturity. **Fig. S7.** Stem starch content across IRRI experiments at A) anthesis and B) maturity. **Fig S8.** Canopy temperature across IRRI drought experiments, grouped by pairs with differing HI under drought but similar biomass and time to flowering. **Fig S9.** NDVI across IRRI drought experiments, grouped by pairs with differing HI under drought but similar biomass and time to flowering. **Fig. S10.** Photosynthesis rates at anthesis across experiments under drought stress (S) and well-watered (WW) conditions at A) Patna and B) IRRI. **Fig. S11.** Stomatal conductance at anthesis across experiments under drought stress (S) and well-watered (WW) conditions at A) Patna and B) IRRI. **Fig. S12.** Relationships between plant water status (as indicated by stomatal conductance and canopy temperature) and stem carbohydrate remobilization (as indicated by stem soluble sugar content and mobilization efficiency (ME)) across IRRI experiments. **Fig. S13.** Relationships between plant water status (as indicated by stomatal conductance) and stem carbohydrate remobilization (as indicated by mobilization efficiency (ME)).

**Additional file 2.** **Table S6.** Correlations among traits measured in the experiments at IRRI.

**Additional file 3.** **Table S7.** Correlations among traits measured in the experiments at Patna.

## Data Availability

Data will be made available on the IRRI Dataverse site upon publication.
