# Supplementary material for: Disentangling the Roles of Plant Water Status and Stem Carbohydrate Remobilization on Rice Harvest Index Under Drought
Source: Rice (N Y). 2023 Mar 17;16:14. doi: 10.1186/s12284-023-00631-6 (PMC10023821; doi:10.1186/s12284-023-00631-6)
Supplement: Supplementary file 1 — Additional file 1. Table S1. Climatic conditions across each season of study. Table S2. Differences in agronomic traits between genotypes in each pair selected for this study. Table S3. Variance explained by each principal component (PC) from the principal component analysis from Patna and IRRI. Table S4. Mean seasonal canopy temperature differences (IRRI drought stress experiments) grouped by pairs of genotypes with differing HI under drought but similar biomass and time to flowering. Table S5. Mean seasonal NDVI differences (IRRI drought stress experiments) grouped by pairs of genotypes with differing HI under drought but similar biomass and time to flowering. Fig. S1. Genotype Pair 4 did not exhibit the expected HI differences across trials for which the genotypes were selected. Fig. S2. Straw biomass across experiments under drought stress (S) and well-watered (WW) conditions at A) Patna and B) IRRI. Fig. S3. ME ((Stem mass anthesis -Stem mass maturity)/Stem mass anthesis ×100) across experiments at IRRI at ICAR-Patna under A) drought stress and B) well-watered conditions. Fig. S4. Stem soluble sugar content throughout the season in drought (A-B) and well-watered (C-D) experiments at IRRI. Fig. S5. Stem starch content throughout the season in drought (A-B) and well-watered (C-D) experiments at IRRI. Fig. S6. Stem soluble sugar content across IRRI experiments at A) anthesis and B) maturity. Fig. S7. Stem starch content across IRRI experiments at A) anthesis and B) maturity. Fig S8. Canopy temperature across IRRI drought experiments, grouped by pairs with differing HI under drought but similar biomass and time to flowering. Fig S9. NDVI across IRRI drought experiments, grouped by pairs with differing HI under drought but similar biomass and time to flowering. Fig. S10. Photosynthesis rates at anthesis across experiments under drought stress (S) and well-watered (WW) conditions at A) Patna and B) IRRI. Fig. S11. Stomatal conductance at anthesis across experiments u [file 12284_2023_631_MOESM1_ESM.docx]

**Dwivedi, Kumar et al.**

**Disentangling the roles of plant water status and stem carbohydrate remobilization on rice harvest index under drought**

**Supplemental Files**

Table S1. Climatic conditions across each season of study. All IRRI drought experiments were conducted using an automated rainout shelter and therefore no water from rainfall affected those experiments.

| **Patna** | **TMAX (°C)** | **TMIN (°C)** | **TMEAN (°C)** | **RH (%)** | **RAINFALL (mm)** | **RAINFALL- drought treatment (mm)** |
| --- | --- | --- | --- | --- | --- | --- |
| **2017WS** | 31.3 | 23.8 | 27.6 | 88.1 | 531 | 61.1 |
| **2018WS** | 31.1 | 23.0 | 27.1 | 89.2 | 525 | 70.6 |
| **2019WS** | 31.4 | 24.2 | 27.8 | 87.4 | 722 | 372 |
|  |  |  |  |  |  |  |

| **IRRI** | **TMAX (°C)** | **TMIN (°C)** | **TMEAN (°C)** | **RH (%)** | **RAINFALL (mm)** |  |
| --- | --- | --- | --- | --- | --- | --- |
| **2017WS** | 32.2 | 24.6 | 28.4 | 84.7 | 1071 |  |
| **2018DS** | 31.1 | 23.6 | 27.4 | 82.1 | 371 |  |
| **2018WS** | 31.7 | 24.2 | 28.0 | 85.7 | 666 |  |
| **2019DS** | 30.4 | 19.3 | 29.6 | 86.0 | 610 |  |

Table S2. Differences in agronomic traits between genotypes in each pair selected for this study. Values shown are BLUEs calculated across drought stress and well-watered trials at Patna and IRRI, and letter groups indicate significantly different genotypes as determined by emmeans and Tukey’s test.

|  |  | IRRI drought | |  | IRRI Well-watered | | | Patna drought | |  | Patna WW | |
| --- | --- | --- | --- | --- | --- | --- | --- | --- | --- | --- | --- | --- |
| DTF |  |  |  |  |  |  |  |  |  |  |  |  |
| Pair | Genotype | BLUEs | Group |  | BLUEs | Group |  | BLUEs | Group |  | BLUEs | Group |
| 1 | Jabor Sail | 79.2 | ab |  | 80.2 | abc |  | 76.1 | cde |  | 77.0 | cde |
| 1 | Tchampa | 83.4 | ab |  | 78.4 | abc |  | 80.3 | abc |  | 83.1 | ab |
| 2 | Dular | 77.2 | b |  | 74.9 | bc |  | 77.8 | abcd |  | 77.1 | cde |
| 2 | Santhi Sufaid 207 | 81.2 | ab |  | 78.5 | abc |  | 78.2 | abcd |  | 80.2 | abc |
| 3 | Camponi Sml | 76.5 | ab |  | 73.3 | c |  | 73.8 | de |  | 74.8 | de |
| 3 | Gul Murali | 85.6 | ab |  | 77.3 | bc |  | 72.4 | e |  | 73.1 | e |
| 4 | ARC 10955 | 78.6 | ab |  | 73.0 | bc |  | 72.6 | e |  | 72.9 | e |
| 4 | Soloi | 79.2 | ab |  | 80.3 | abc |  | 76.7 | bcde |  | 79.4 | abc |
| 5 | E Zi 124 |  |  |  | 86.6 | a |  | 81.1 | ab |  | 83.7 | a |
| 5 | Wanni Dahanala | 80.3 | ab |  | 77.9 | abc |  | 78.4 | abc |  | 79.1 | bcd |
| 6 | Aus 257 | 77.1 | ab |  | 75.7 | bc |  | 78.0 | abcd |  | 79.3 | abc |
| 6 | DZ78 | 78.1 | ab |  | 78.4 | abc |  | 78.8 | abc |  | 81.5 | abc |
| 7 | IR64 | 86.7 | a |  | 81.3 | ab |  | 81.6 | a |  | 83.0 | ab |
| 7 | IR74371-70-1-1 | 81.5 | ab |  | 79.6 | abc |  | 80.0 | abc |  | 82.5 | ab |
|  |  |  |  |  |  |  |  |  |  |  |  |  |
| Plant Height (cm) | |  |  |  |  |  |  |  |  |  |  |  |
| Pair | Genotype | BLUEs | Group |  | BLUEs | Group |  | BLUEs | Group |  | BLUEs | Group |
| 1 | Jabor Sail | 105.4 | ab |  | 126.4 | abcd |  | 128.3 | ab |  | 150.5 | abc |
| 1 | Tchampa | 104.1 | ab |  | 147.4 | a |  | 120.7 | abc |  | 165.0 | a |
| 2 | Dular | 101.3 | ab |  | 131.9 | ab |  | 129.5 | ab |  | 141.7 | bcd |
| 2 | Santhi Sufaid 207 | 88.1 | bc |  | 125.8 | abcd |  | 121.0 | abc |  | 132.8 | cde |
| 3 | Camponi Sml | 99.7 | ab |  | 128.8 | abc |  | 123.3 | ab |  | 140.7 | bcd |
| 3 | Gul Murali | 91.9 | abc |  | 140.2 | a |  | 119.6 | bc |  | 132.9 | cde |
| 4 | ARC 10955 | 92.0 | abc |  | 122.0 | abcd |  | 126.6 | ab |  | 123.4 | def |
| 4 | Soloi | 100.4 | ab |  | 117.9 | abcd |  | 130.1 | ab |  | 144.0 | bc |
| 5 | E Zi 124 |  |  |  | 93.9 | d |  | 135.7 | ab |  | 158.0 | ab |
| 5 | Wanni Dahanala | 87.0 | abc |  | 128.4 | abc |  | 119.8 | abc |  | 140.9 | bcd |
| 6 | Aus 257 | 106.7 | a |  | 138.5 | a |  | 142.2 | a |  | 156.6 | ab |
| 6 | DZ78 | 101.4 | ab |  | 121.1 | abcd |  | 135.9 | ab |  | 151.6 | abc |
| 7 | IR64 | 63.0 | d |  | 104.0 | cd |  | 95.2 | d |  | 106.1 | f |
| 7 | IR74371-70-1-1 | 74.4 | cd |  | 106.4 | bcd |  | 99.5 | cd |  | 118.3 | ef |
|  |  |  |  |  |  |  |  |  |  |  |  |  |
| Tiller # (m^-2^) | |  |  |  |  |  |  |  |  |  |  |  |
| Pair | Genotype | BLUEs | Group |  | BLUEs | Group |  | BLUEs | Group |  | BLUEs | Group |
| 1 | Jabor Sail | 236.9 | a |  | 235.2 | a |  | 217.5 | ab |  | 236.8 | a |
| 1 | Tchampa | 219.4 | a |  | 304.3 | a |  | 185.3 | cde |  | 208.9 | a |
| 2 | Dular | 220.0 | a |  | 295.7 | a |  | 208.3 | abc |  | 239.8 | a |
| 2 | Santhi Sufaid 207 | 223.3 | a |  | 305.5 | a |  | 181.5 | cde |  | 216.2 | a |
| 3 | Camponi Sml | 255.2 | a |  | 247.4 | a |  | 230.9 | a |  | 240.4 | a |
| 3 | Gul Murali | 266.1 | a |  | 254.3 | a |  | 202.4 | abcd |  | 210.1 | a |
| 4 | ARC 10955 | 247.7 | a |  | 275 | a |  | 205.1 | abc |  | 200.5 | a |
| 4 | Soloi | 203.2 | a |  | 275.7 | a |  | 190.5 | bcde |  | 220.1 | a |
| 5 | E Zi 124 |  |  |  | 404.3 | a |  | 208.6 | abc |  | 216.0 | a |
| 5 | Wanni Dahanala | 171.1 | a |  | 342.4 | a |  | 180.4 | cde |  | 216.3 | a |
| 6 | Aus 257 | 348.3 | a |  | 405.4 | a |  | 161.3 | e |  | 191.6 | a |
| 6 | DZ78 | 362.3 | a |  | 321.7 | a |  | 210.8 | abc |  | 229.7 | a |
| 7 | IR64 | 254.6 | a |  | 321.7 | a |  | 174.0 | de |  | 200.4 | a |
| 7 | IR74371-70-1-1 | 248.3 | a |  | 300.8 | a |  | 224.7 | a |  | 227.5 | a |
|  |  |  |  |  |  |  |  |  |  |  |  |  |
| Grain yield (g m^-2^) | |  |  |  |  |  |  |  |  |  |  |  |
| Pair | Genotype | BLUEs | Group |  | BLUEs | Group |  | BLUEs | Group |  | BLUEs | Group |
| 1 | Jabor Sail | 86.3 | ab |  | 246.4 | a |  | 232.1 | a |  | 302.17 | c |
| 1 | Tchampa | 76.1 | ab |  | 403.3 | a |  | 248.5 | a |  | 380.02 | abc |
| 2 | Dular | 134.2 | a |  | 249.9 | a |  | 242.4 | a |  | 306.38 | c |
| 2 | Santhi Sufaid 207 | 63.6 | ab |  | 249.0 | a |  | 218.2 | a |  | 410.68 | abc |
| 3 | Camponi Sml | 128.1 | a |  | 255.8 | a |  | 257.5 | a |  | 313.68 | bc |
| 3 | Gul Murali | 0.0 | b |  | 264.7 | a |  | 219.1 | a |  | 380.23 | abc |
| 4 | ARC 10955 | 50.3 | ab |  | 153.0 | a |  | 233.5 | a |  | 291.38 | c |
| 4 | Soloi | 100.6 | ab |  | 215.8 | a |  | 229.3 | a |  | 365.28 | abc |
| 5 | E Zi 124 |  |  |  | 284.4 | a |  | 195.5 | a |  | 372.78 | abc |
| 5 | Wanni Dahanala | 47.5 | ab |  | 367.9 | a |  | 299.5 | a |  | 416.06 | abc |
| 6 | Aus 257 | 127.3 | a |  | 298.3 | a |  | 266.1 | a |  | 348.54 | abc |
| 6 | DZ78 | 89.1 | ab |  | 199.8 | a |  | 222.4 | a |  | 370.86 | abc |
| 7 | IR64 | 8.2 | b |  | 344.1 | a |  | 231.8 | a |  | 498.73 | ab |
| 7 | IR74371-70-1-1 | 29.5 | b |  | 325.4 | a |  | 327.8 | a |  | 506.09 | a |

Table S3.Variance explained by each principal component (PC) from the principal component analysis from Patna and IRRI.

| Patna PCA |  |  |  |  |  |  |  |
| --- | --- | --- | --- | --- | --- | --- | --- |
|  | PC1 | PC2 | PC3 | PC4 | PC5 | PC6 | PC7 |
| Standard deviation | 1.4763 | 1.3069 | 0.9958 | 0.9545 | 0.80646 | 0.73361 | 0.14513 |
| Proportion of Variance | 0.3114 | 0.244 | 0.1417 | 0.1302 | 0.09291 | 0.07688 | 0.00301 |
| Cumulative Proportion | 0.3114 | 0.5554 | 0.697 | 0.8272 | 0.92011 | 0.99699 | 1 |
|  |  |  |  |  |  |  |  |
| IRRI PCA |  |  |  |  |  |  |  |
|  | PC1 | PC2 | PC3 | PC4 | PC5 | PC6 | PC7 |
| Standard deviation | 1.8231 | 1.3027 | 0.9543 | 0.75293 | 0.55712 | 0.38229 | 0.21268 |
| Proportion of Variance | 0.4748 | 0.2424 | 0.1301 | 0.08099 | 0.04434 | 0.02088 | 0.00646 |
| Cumulative Proportion | 0.4748 | 0.7172 | 0.8473 | 0.92832 | 0.97266 | 0.99354 | 1 |
|  |  |  |  |  |  |  |  |

Table S4. Mean seasonal canopy temperature differences (IRRI drought stress experiments) grouped by pairs of genotypes with differing HI under drought but similar biomass and time to flowering. Letter groups indicate significantly different genotypes as determined by Tukey’s test, and pairs with significant differences between genotypes are highlighted.

|  |  | p<0.001 |  | p<0.001 |  | p<0.001 |  | p<0.001 |  |
| --- | --- | --- | --- | --- | --- | --- | --- | --- | --- |
| Pair | Genotype | **17WS** | groups | **18DS** | groups | **18WS** | groups | **19DS** | groups |
| 1 | Jabor Sail |  |  |  |  | 34.1 | de | 33.9 | e |
| 1 | Tchampa |  |  |  |  | 35.0 | bcd | 33.5 | e |
| 2 | Dular | 33.8 | e | 32.2 | e | 33.8 | e | 33.9 | e |
| 2 | Santhi Sufaid 207 | 35.0 | bcd | 33.5 | cd | 34.5 | cde | 33.6 | e |
| 3 | Camponi Sml | 33.9 | de | 31.8 | e | 33.9 | e | 34.2 | e |
| 3 | Gul Murali | 34.5 | cde | 32.7 | de | 35.2 | bc | 35.2 | bc |
| 4 | ARC 10955 | 34.5 | cde |  |  | 34.5 | cde | 34.4 | cde |
| 4 | Soloi | 33.7 | e |  |  | 33.7 | e | 34.3 | de |
| 5 | E Zi 124 |  |  | 35.4 | a | 36.9 | a | 36.4 | a |
| 5 | Wanni Dahanala | 35.7 | ab | 33.4 | cd | 35.8 | ab | 35.2 | bcd |
| 6 | Aus 257 | 36.4 | a | 31.9 | e | 33.8 | e | 33.7 | e |
| 6 | DZ78 | 34.1 | cde | 33.7 | bc | 33.6 | e | 33.8 | e |
| 7 | IR64 | 35.9 | ab | 34.6 | ab | 35.9 | ab | 36.2 | a |
| 7 | IR74371-70-1-1 | 35.2 | bc | 33.2 | cd | 35.7 | b | 35.8 | ab |

| Pair | Genotype | **17WS** | groups | **18DS** | groups | **18WS** | groups | **19DS** | groups |
| --- | --- | --- | --- | --- | --- | --- | --- | --- | --- |
| 1 | Jabor Sail |  |  |  |  | 0.446 | ab | 0.472 | b |
| 1 | Tchampa |  |  |  |  | 0.395 | bc | 0.533 | a |
| 2 | Dular | 0.660 | a | 0.544 | a | 0.469 | a | 0.533 | a |
| 2 | Santhi Sufaid 207 | 0.622 | ab | 0.483 | bc | 0.442 | ab | 0.514 | a |
| 3 | Camponi Sml | 0.693 | a | 0.521 | ab | 0.469 | a | 0.467 | b |
| 3 | Gul Murali | 0.698 | a | 0.460 | cd | 0.349 | cde | 0.423 | c |
| 4 | ARC 10955 |  |  |  |  | 0.445 | ab | 0.475 | b |
| 4 | Soloi |  |  |  |  | 0.439 | ab | 0.461 | b |
| 5 | E Zi 124 |  |  | 0.366 | f | 0.300 | e | 0.372 | e |
| 5 | Wanni Dahanala | 0.545 | bc | 0.432 | cde | 0.357 | cd | 0.412 | cd |
| 6 | Aus 257 | 0.367 | d | 0.532 | ab | 0.452 | a | 0.472 | b |
| 6 | DZ78 | 0.621 | ab | 0.424 | de | 0.448 | ab | 0.464 | b |
| 7 | IR64 | 0.475 | c | 0.381 | ef | 0.339 | de | 0.377 | de |
| 7 | IR74371-70-1-1 | 0.493 | c | 0.411 | def | 0.335 | de | 0.407 | cde |

Table S5. Mean seasonal NDVI differences (IRRI drought stress experiments) grouped by pairs of genotypes with differing HI under drought but similar biomass and time to flowering. Letter groups indicate significantly different genotypes as determined by Tukey’s test, and pairs with significant differences between genotypes are highlighted.

Table S6. Correlations among traits measured in the experiments at IRRI.

See attached Excel file.

Table S7. Correlations among traits measured in the experiments at Patna.

See attached Excel file.


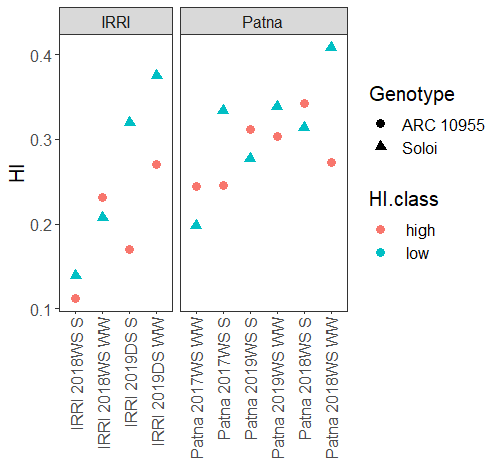


Fig. S1. Genotype Pair 4 did not exhibit the expected HI differences across trials for which the genotypes were selected.


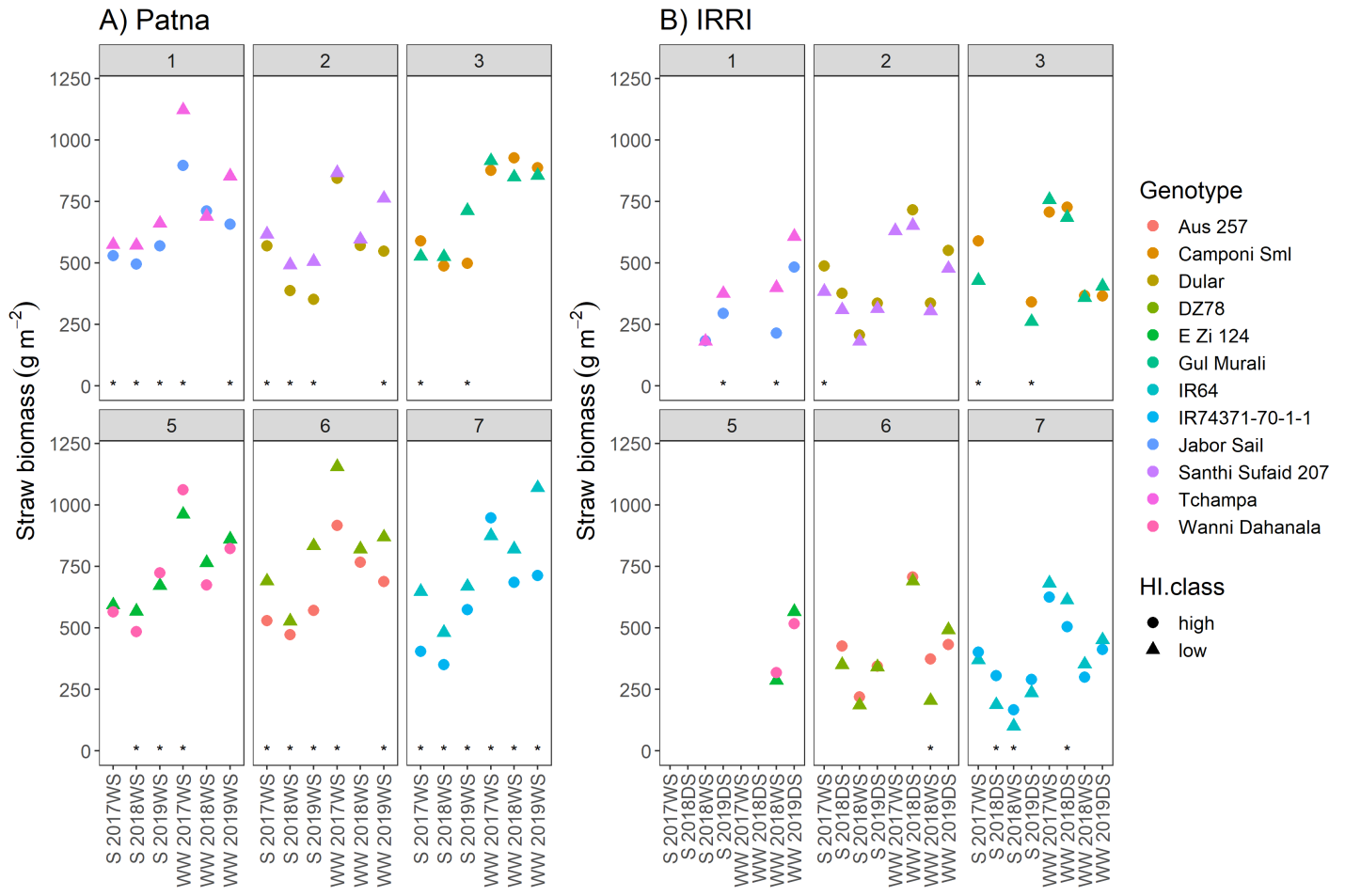


Fig. S2. Straw biomass across experiments under drought stress (S) and well-watered (WW) conditions at A) Patna and B) IRRI. The boxes at the top of each panel indicate the pair ID. Significant differences between genotypes within a pair (based on different letter groups in according to Tukey’s test) are indicated by *. WS: wet season, DS: dry season.


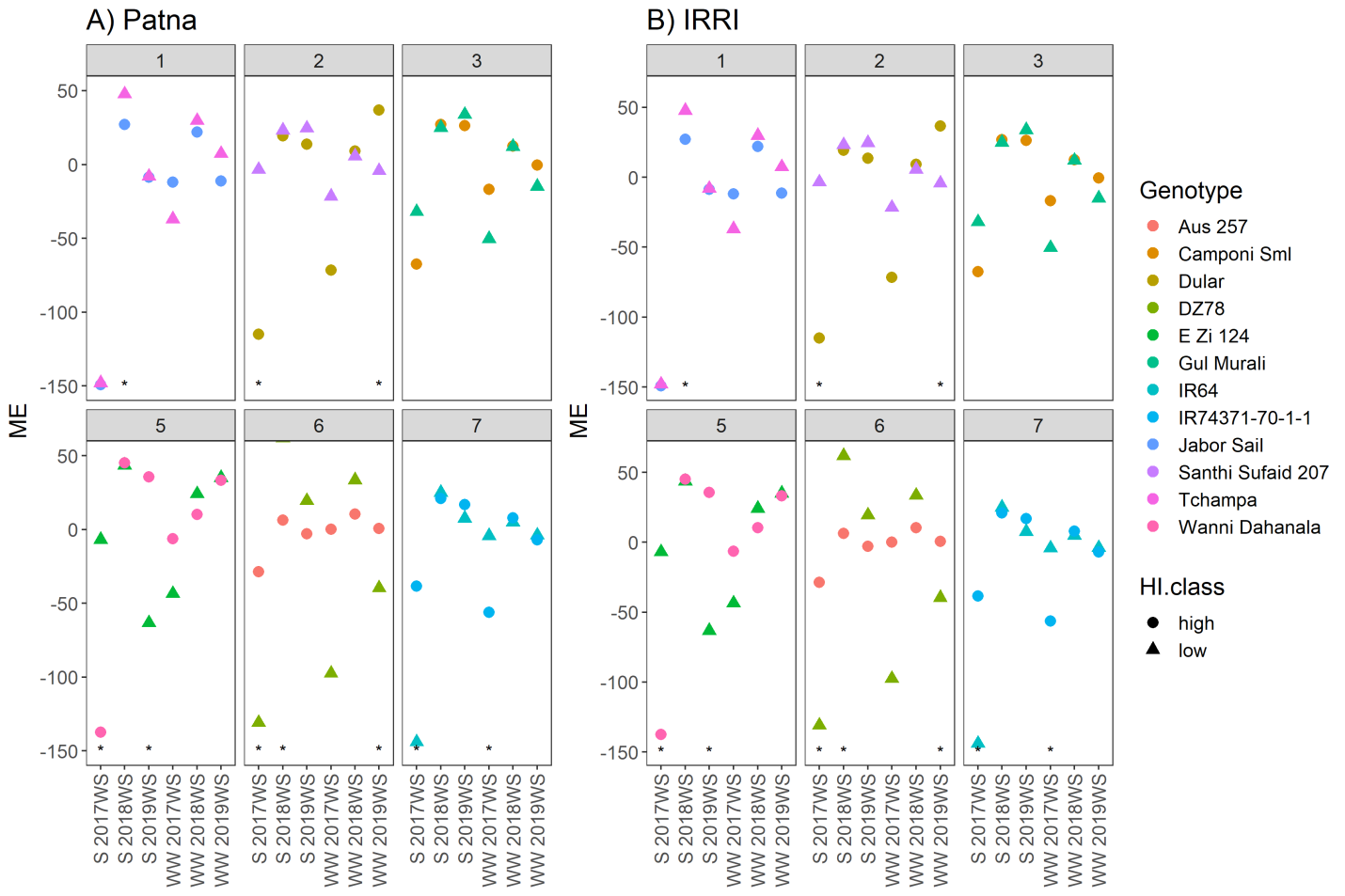


Fig. S3. ME ($\frac{Stem mass anthesis -Stem mass maturity}{Stem mass anthesis}$ $\times100$) across experiments at IRRI at ICAR-Patna under A) drought stress and B) well-watered conditions. The boxes at the top of each panel indicate the pair ID.


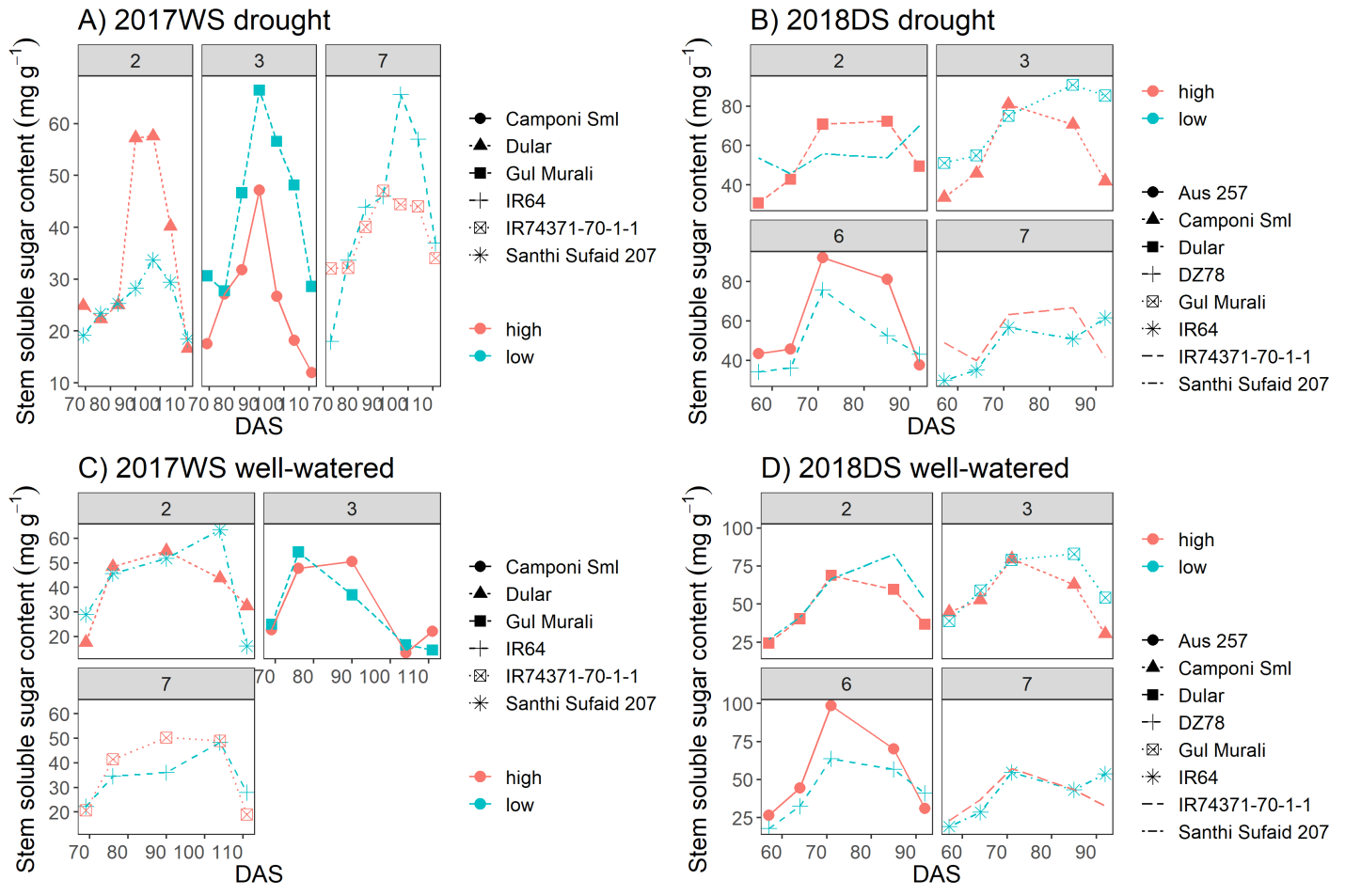


Fig. S4. Stem soluble sugar content throughout the season in drought (A-B) and well-watered (C-D) experiments at IRRI. Pairs of genotypes are shown that were selected for differing HI under drought but similar biomass and time to flowering. The boxes at the top of each panel indicate the pair ID (see Table 1 for the genotypes in each pair). DAS: days after sowing. Letter groups indicate significant differences among genotypes within each trial according to the LSD test.


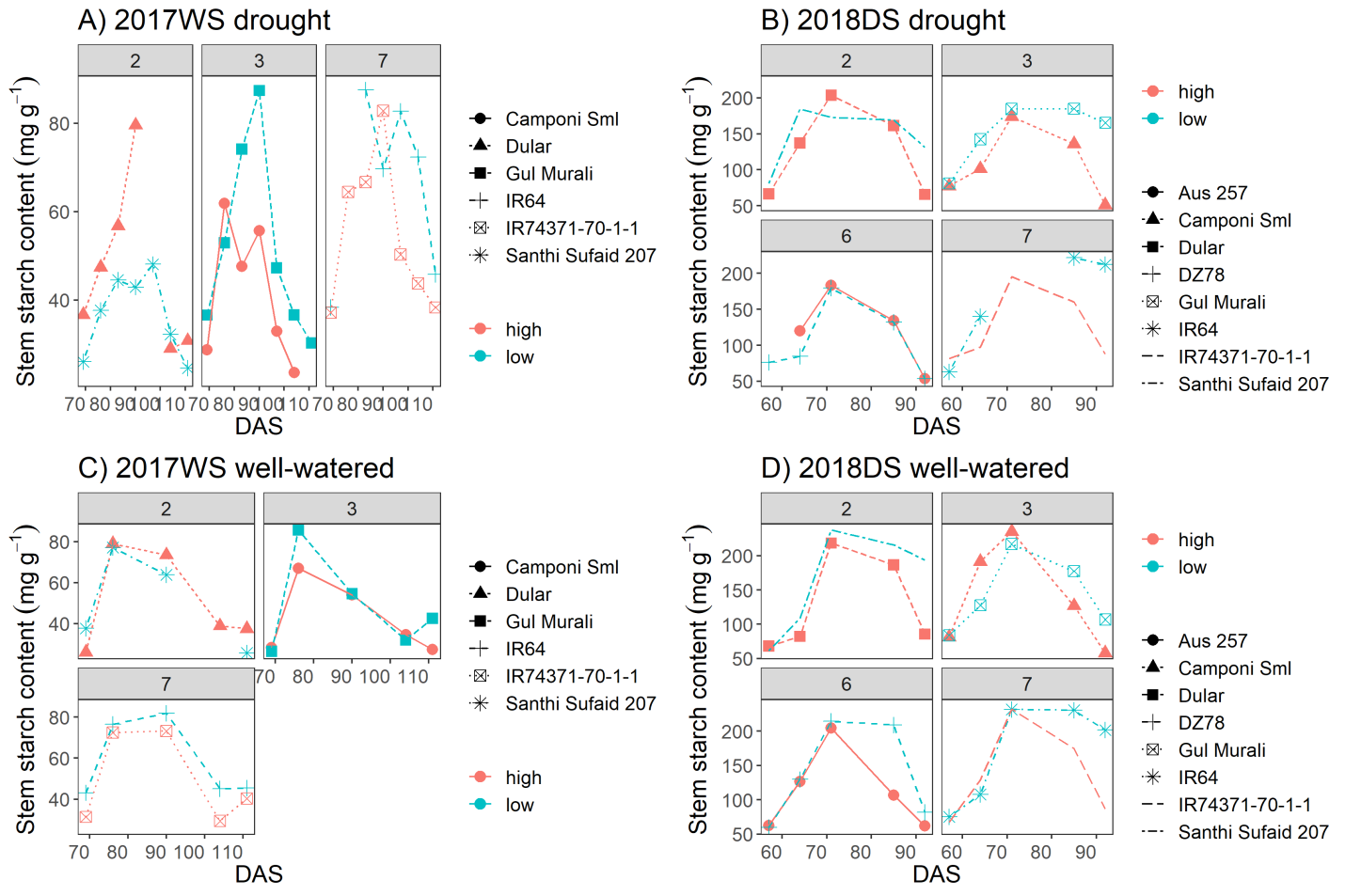


Fig. S5. Stem starch content throughout the season in drought (A-B) and well-watered (C-D) experiments at IRRI. Pairs of genotypes are shown that were selected for differing HI under drought but similar biomass and time to flowering. The boxes at the top of each panel indicate the pair ID (see Table 1 for the genotypes in each pair). DAS: days after sowing. Letter groups indicate significant differences among genotypes within each trial according to the LSD test.


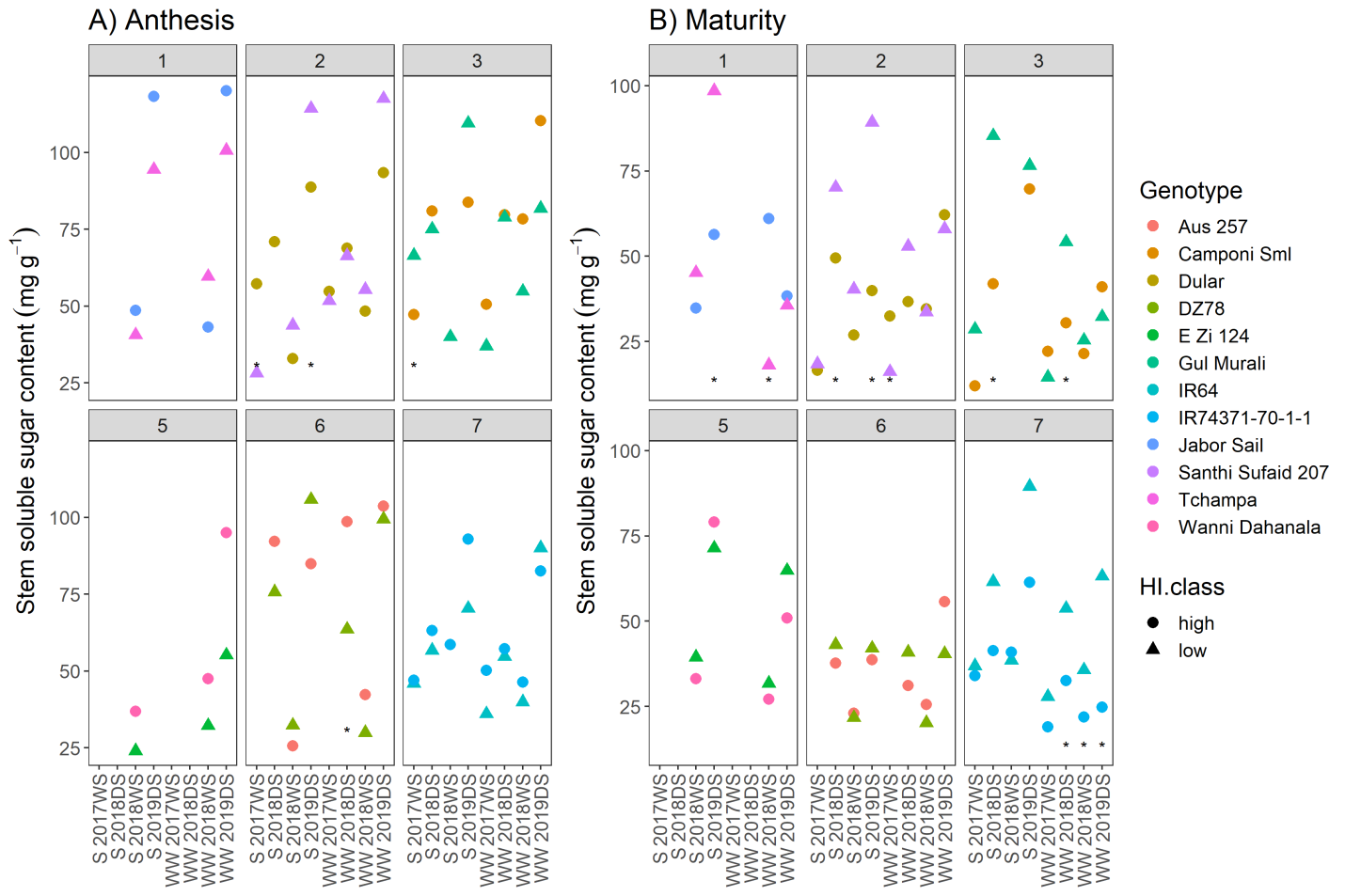


Fig. S6. Stem soluble sugar content across IRRI experiments at A) anthesis and B) maturity. The boxes at the top of each panel indicate the pair ID. Significant differences between genotypes within a pair (based on different letter groups in according to Tukey’s test) are indicated by *. WS: wet season, DS: dry season.


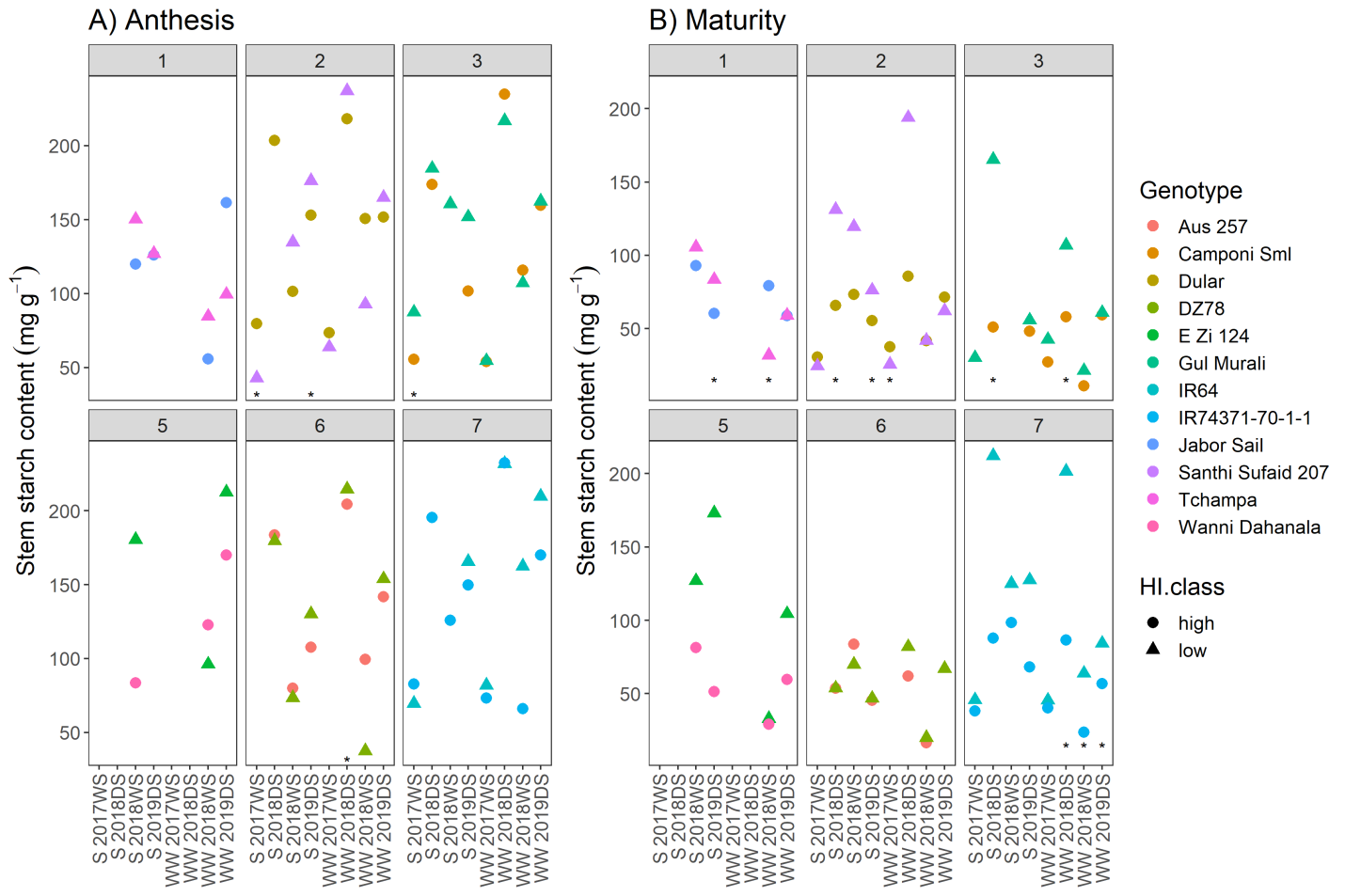


Fig. S7. Stem starch content across IRRI experiments at A) anthesis and B) maturity. The boxes at the top of each panel indicate the pair ID. Significant differences between genotypes within a pair (based on different letter groups in according to Tukey’s test) are indicated by *. WS: wet season, DS: dry season.


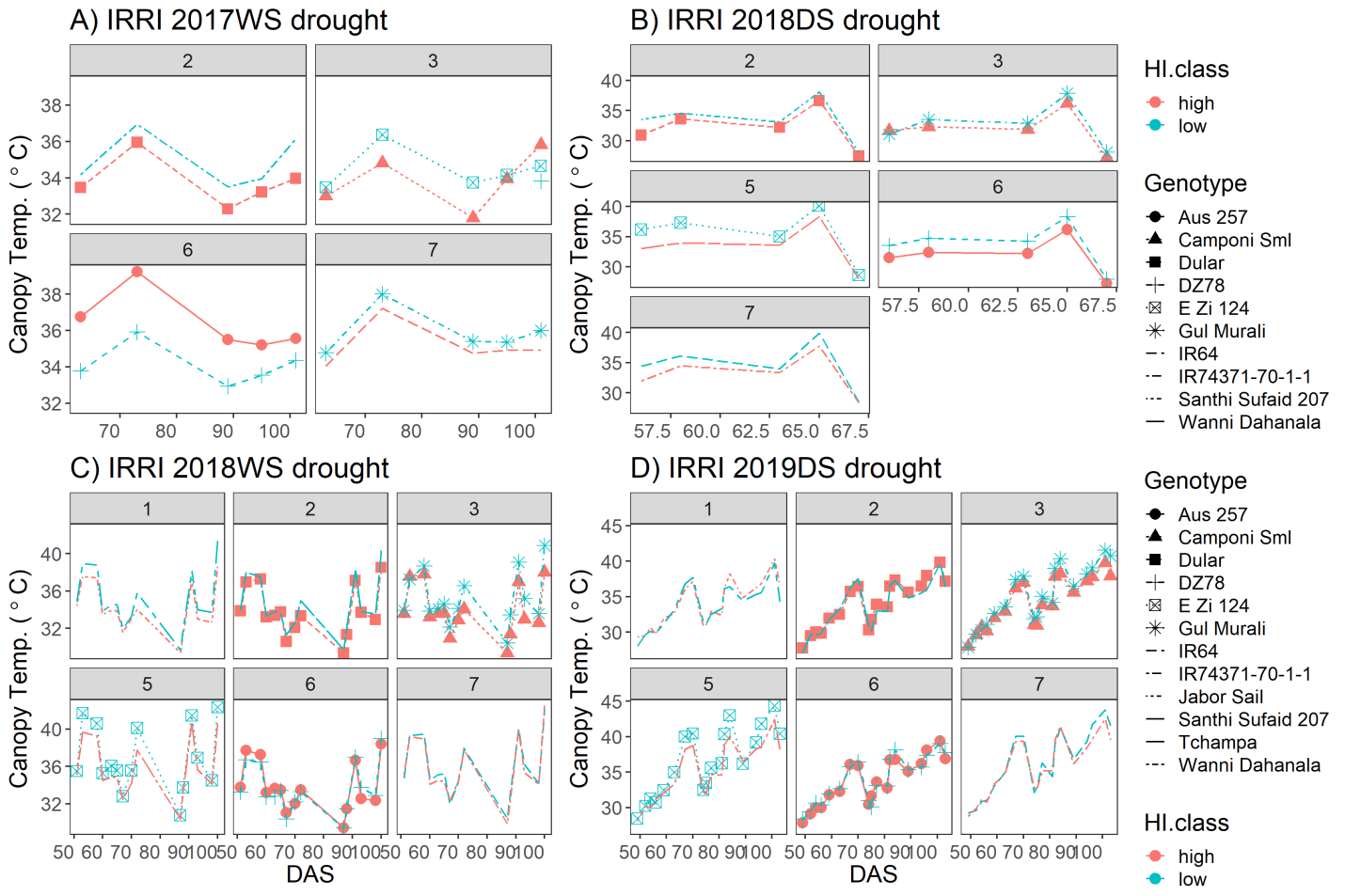


Fig S8. Canopy temperature across IRRI drought experiments, grouped by pairs with differing HI under drought but similar biomass and time to flowering. The boxes at the top of each panel indicate the pair ID. Significant differences in mean seasonal canopy temperature are indicated in Table 4. DAS: days after sowing. Mean seasonal canopy temperature values are shown in Table S8.


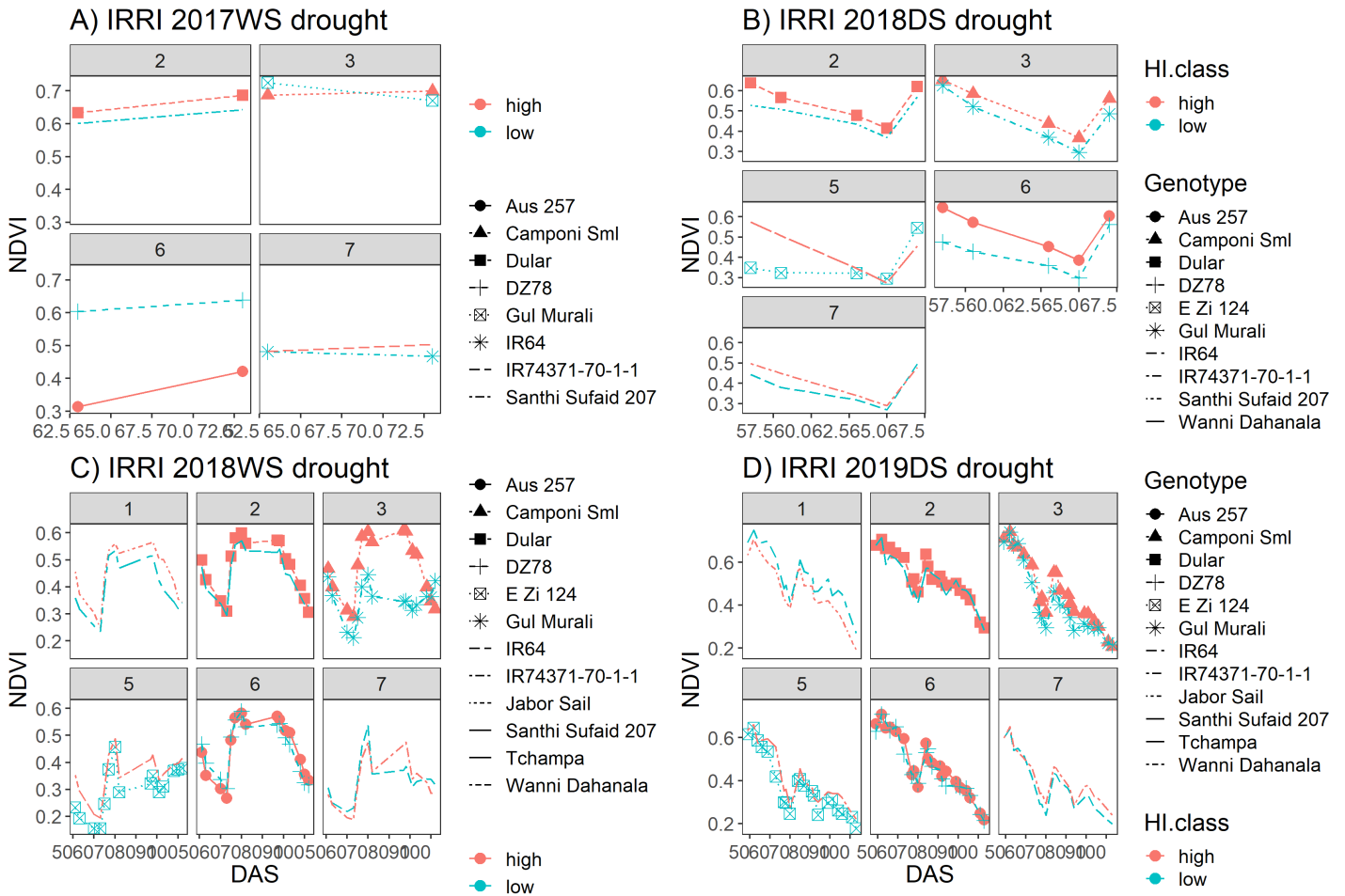


Fig S9. NDVI across IRRI drought experiments, grouped by pairs with differing HI under drought but similar biomass and time to flowering. The boxes at the top of each panel indicate the pair ID. Significant differences in mean seasonal canopy temperature are indicated in Table 4. DAS: days after sowing. Mean seasonal NDVI values are shown in Table S9.


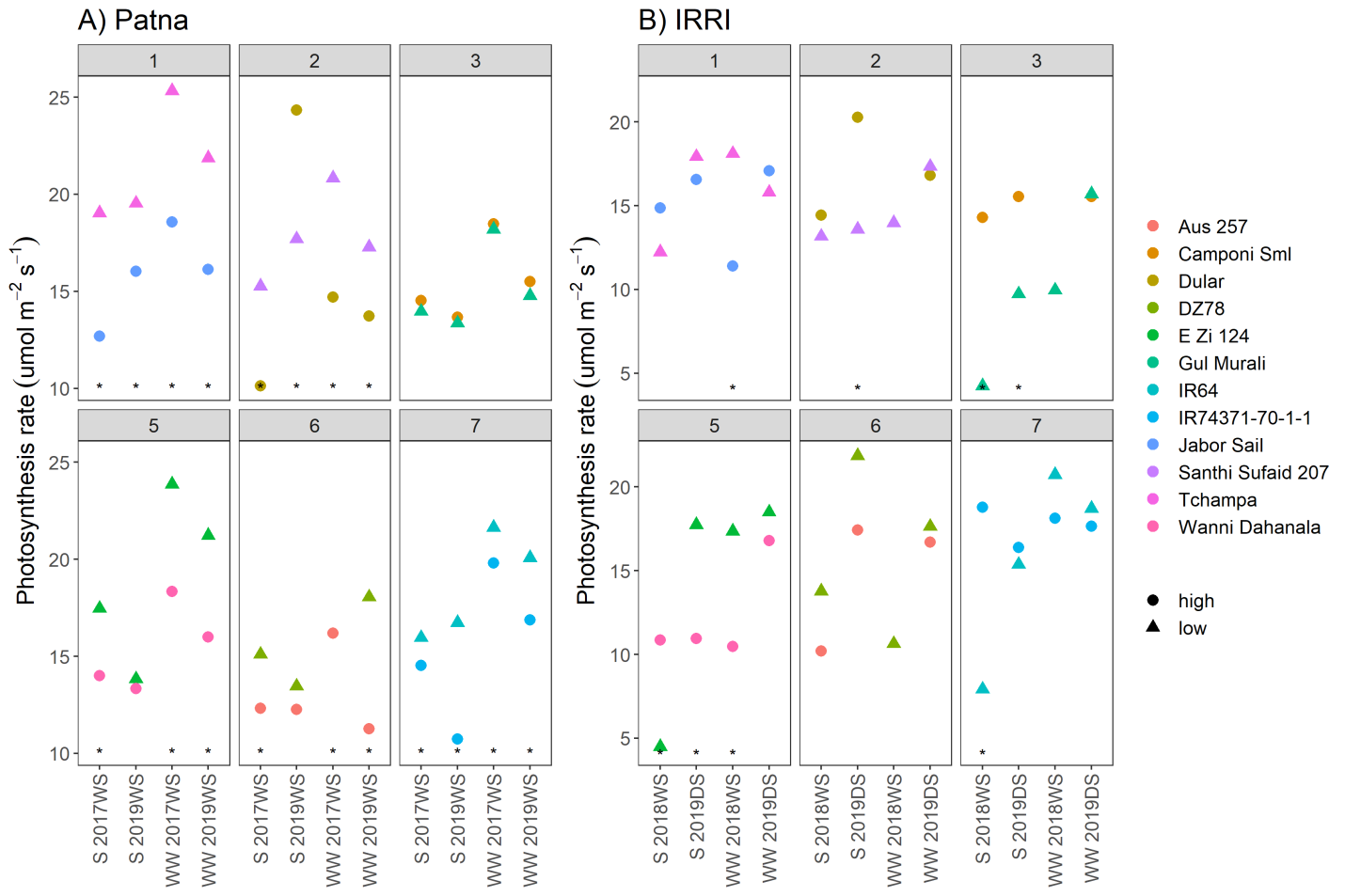


Fig. S10. Photosynthesis rates at anthesis across experiments under drought stress (S) and well-watered (WW) conditions at A) Patna and B) IRRI. The boxes at the top of each panel indicate the pair ID. Significant differences between genotypes within a pair (based on different letter groups in according to Tukey’s test) are indicated by *. WS: wet season, DS: dry season.


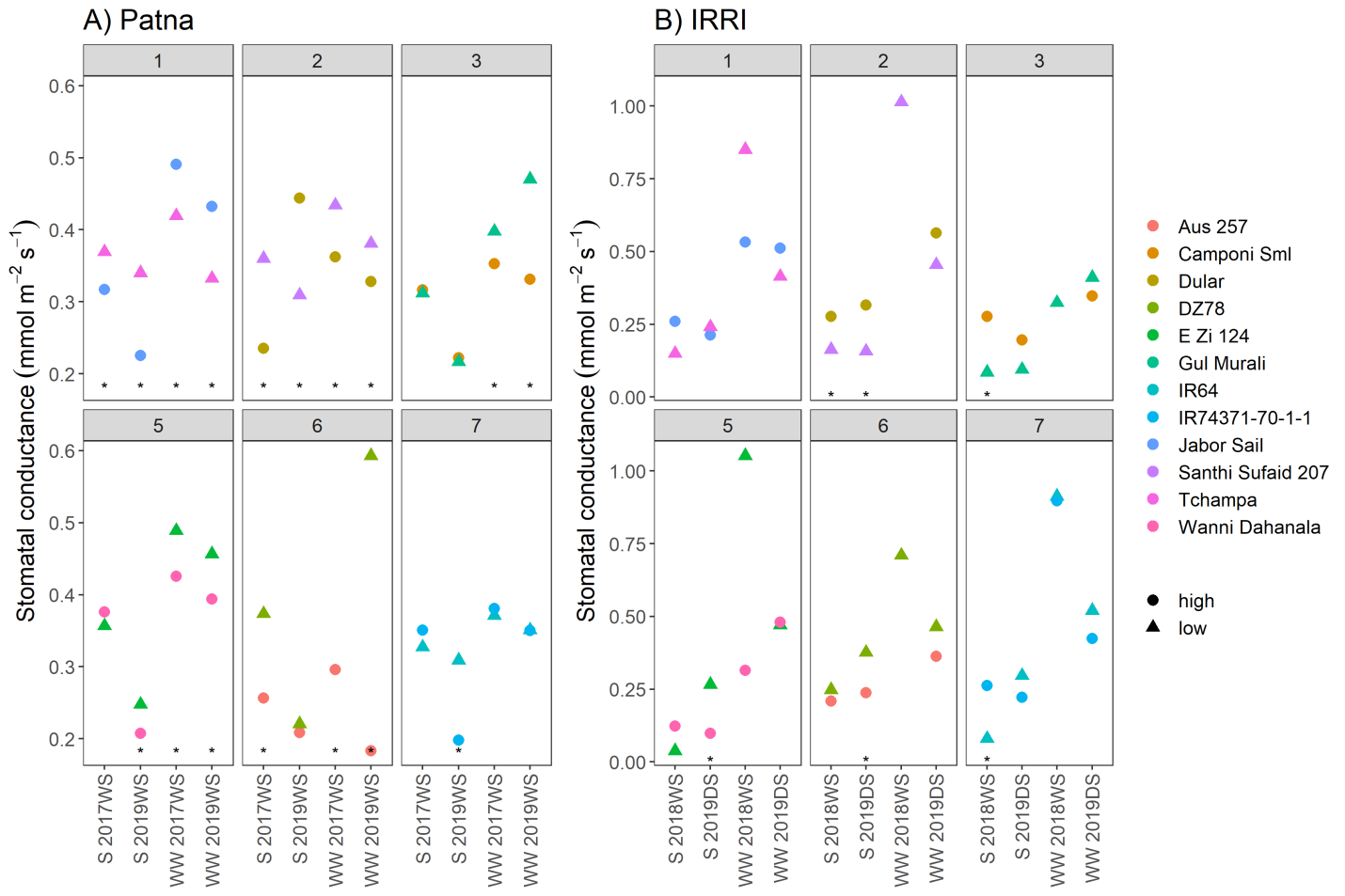


Fig. S11. Stomatal conductance at anthesis across experiments under drought stress (S) and well-watered (WW) conditions at A) Patna and B) IRRI. The boxes at the top of each panel indicate the pair ID. Significant differences between genotypes within a pair (based on different letter groups in according to Tukey’s test) are indicated by *. WS: wet season, DS: dry season.


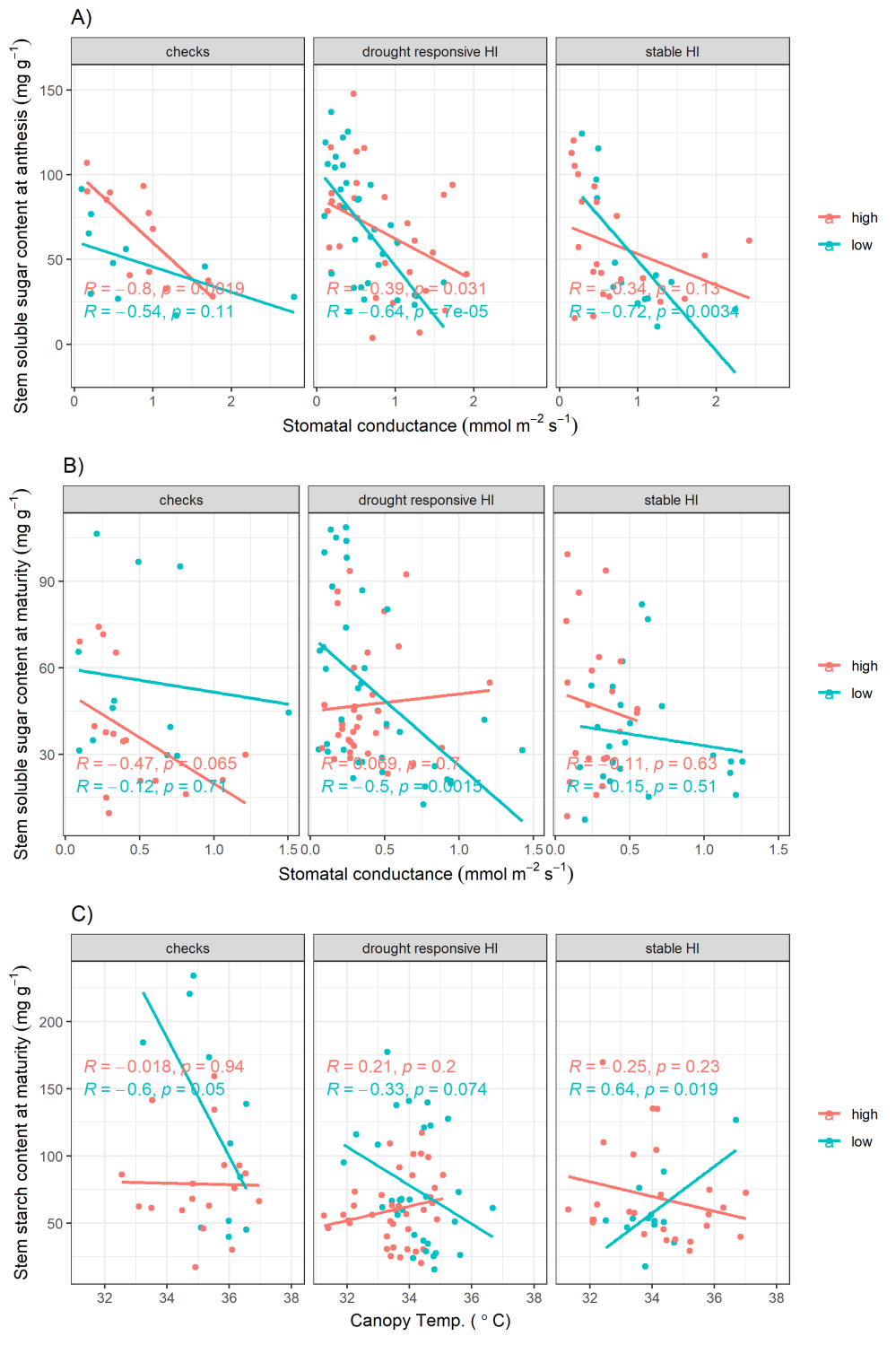


Fig. S12. Relationships between plant water status (as indicated by stomatal conductance and canopy temperature) and stem carbohydrate remobilization (as indicated by stem soluble sugar content and mobilization efficiency (ME)) across IRRI experiments. Correlations are shown between A) stomatal conductance (measured all on the same date) and stem soluble sugar levels at anthesis, B) between stomatal conductance (measured at anthesis for each genotype) and stem soluble sugar levels at anthesis, and C) between canopy temperature in the IRRI drought experiments and ME. Groups whose difference in harvest index between high-HI and low-HI genotype pairs appeared to be drought-responsive (Pairs 3 and 5) or stable (Pairs 1, 2, and 6) are compared. The checks were IR74371-70-1-1 (high-HI) and IR64 (low HI).


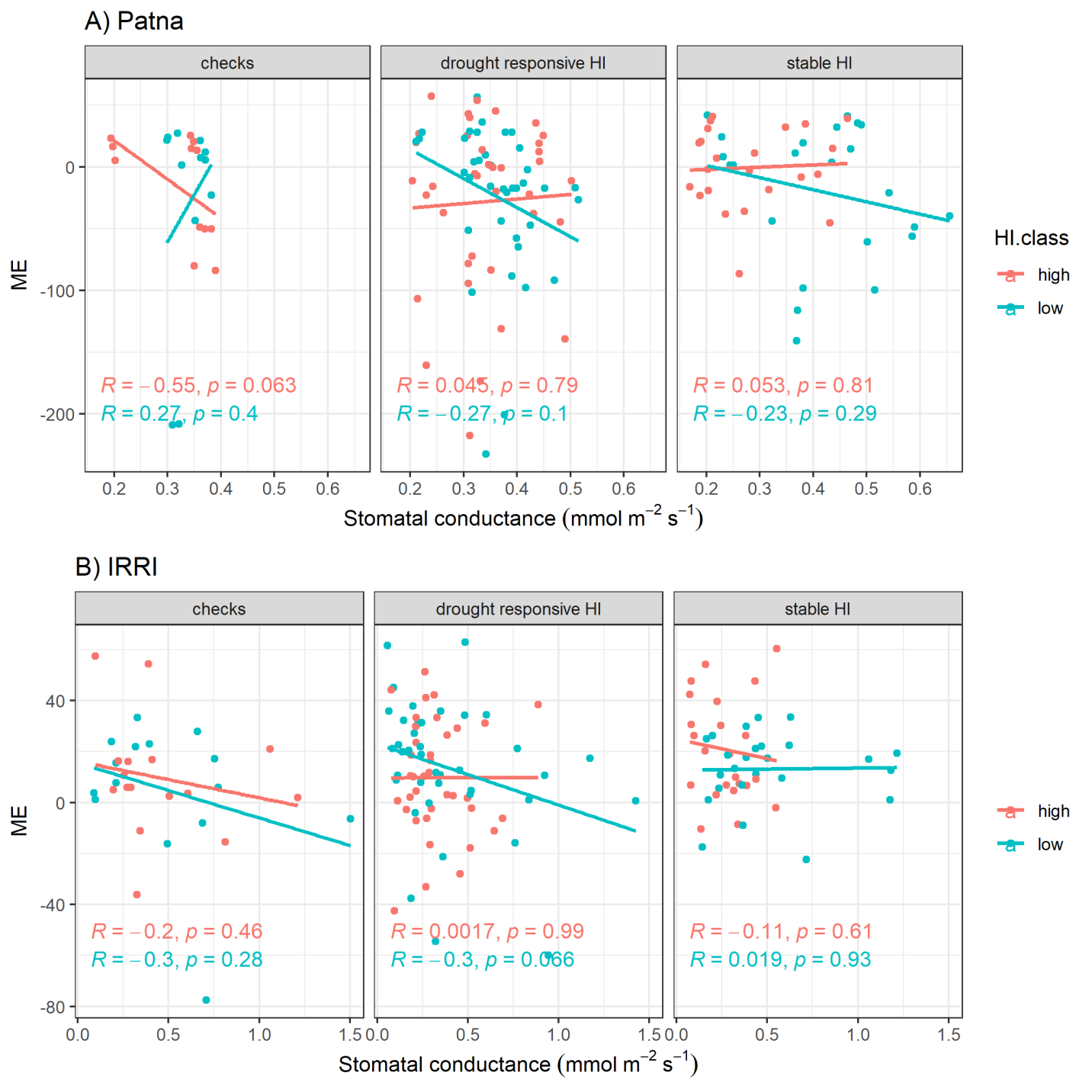


Fig. S13. Relationships between plant water status (as indicated by stomatal conductance) and stem carbohydrate remobilization (as indicated by mobilization efficiency (ME)). Correlations are shown between stomatal conductance (measured at anthesis for each genotype) and ME at A) Patna and B) IRRI. Groups whose difference in harvest index between high-HI and low-HI genotype pairs appeared to be drought-responsive (Pairs 3 and 5) or stable (Pairs 1, 2, and 6) are compared. The checks were IR74371-70-1-1 (high-HI) and IR64 (low HI).
